# Does Increased Exercise or Physical Activity Alter Ad-Libitum Daily Energy Intake or Macronutrient Composition in Healthy Adults? A Systematic Review

**DOI:** 10.1371/journal.pone.0083498

**Published:** 2014-01-15

**Authors:** Joseph E. Donnelly, Stephen D. Herrmann, Kate Lambourne, Amanda N. Szabo, Jeffery J. Honas, Richard A. Washburn

**Affiliations:** Cardiovascular Research Institute, Center for Physical Activity and Weight Management, University of Kansas Medical Center, Kansas City, Kansas, United States of America; Universidad Pablo de Olavide, Centro Andaluz de Biología del Desarrollo-CSIC, Spain

## Abstract

**Background:**

The magnitude of the negative energy balance induced by exercise may be reduced due to compensatory increases in energy intake.

**Objective:**

To address the question: Does increased exercise or physical activity alter ad-libitum daily energy intake or macronutrient composition in healthy adults?

**Data Sources:**

PubMed and Embase were searched (January 1990–January 2013) for studies that presented data on energy and/or macronutrient intake by level of exercise, physical activity or change in response to exercise. Ninety-nine articles (103 studies) were included.

**Study Eligibility Criteria:**

Primary source articles published in English in peer-reviewed journals. Articles that presented data on energy and/or macronutrient intake by level of exercise or physical activity or changes in energy or macronutrient intake in response to acute exercise or exercise training in healthy (non-athlete) adults (mean age 18–64 years).

**Study Appraisal and Synthesis Methods:**

Articles were grouped by study design: cross-sectional, acute/short term, non-randomized, and randomized trials. Considerable heterogeneity existed within study groups for several important study parameters, therefore a meta-analysis was considered inappropriate. Results were synthesized and presented by study design.

**Results:**

No effect of physical activity, exercise or exercise training on energy intake was shown in 59% of cross-sectional studies (n = 17), 69% of acute (n = 40), 50% of short-term (n = 10), 92% of non-randomized (n = 12) and 75% of randomized trials (n = 24). Ninety-four percent of acute, 57% of short-term, 100% of non-randomized and 74% of randomized trials found no effect of exercise on macronutrient intake. Forty-six percent of cross-sectional trials found lower fat intake with increased physical activity.

**Limitations:**

The literature is limited by the lack of adequately powered trials of sufficient duration, which have prescribed and measured exercise energy expenditure, or employed adequate assessment methods for energy and macronutrient intake.

**Conclusions:**

We found no consistent evidence that increased physical activity or exercise effects energy or macronutrient intake.

## Introduction

Data from the 2009–2010 National Health and Nutrition Examination Survey (NHANES) suggest that 68.8% of those age ≥20 years are overweight (Body Mass Index [BMI]≥25 kg/m^2^) while 35.7% are obese (BMI≥30 kg/m^2^) [1] with approximately 51% of US adults predicted to be obese by 2030 Finkelstein [2]. Medical expenditures associated with the treatment of obesity and obesity related conditions are estimated at greater than $147 billion annually [2]. Data from the NHANES (2003–2008) indicated that among adults (18–54 years) approximately 75% of women and 54% of men expressed a desire to lose weight while 61% of women and 39% of men were actively pursuing weight control [3].

Exercise is recommended for weight management by several governmental agencies and professional organizations including the Association for the Study of Obesity [4], the Institute of Medicine [5], the U.S. Federal guidelines on physical activity [6], Healthy People 2020 [7] and the American College of Sports Medicine [8]. Compared with weight loss induced by energy restriction, weight loss achieved by exercise is composed predominantly of fat mass, while fat-free mass is preserved [9]–[11] and resting metabolic rate (RMR) is generally unchanged [12], [13], or slightly increased [14], [15], factors that may be associated with improved long term weight loss maintenance. However, several reports have demonstrated that the accumulated energy balance induced by an exercise intervention alone produces less of a negative energy balance than theoretically predicted for the imposed level of exercise-induced energy expenditure [16]–[18]. The energy balance induced by exercise training may be reduced due to compensatory changes in energy intake, non-exercise physical activity, or both [19]–[21]; thereby reducing the magnitude of observed weight loss. Although several narrative reviews regarding the impact of exercise on energy intake and appetite hormones have been conducted [22]–[29], we are aware of only one systematic review/meta-analysis on this topic. Schubert *et al.* [30] recently published a meta-analysis on the effect of acute exercise on subsequent energy intake that included only studies that assessed energy intake for ≤24 hours post-exercise in healthy (lean and/or obese), non-smoking individuals. To date, no systematic reviews on the effect of exercise and energy and macronutrient intake have been conducted that evaluated both the effects of acute exercise and exercise training and have included data from studies utilizing a variety of designs, e.g. cross-sectional, acute-crossover, non-randomized and randomized trials. Therefore, the aim of this systematic review was to identify and evaluate studies that have employed a variety of designs to assess the impact of both acute exercise and exercise training on energy and macronutrient intake. Results of this review will clarify our understanding of the association between exercise and energy intake and identify both exercise parameters including mode, frequency, intensity and duration and participant characteristics including age, gender, body weight, activity level that may impact this association. Such information will be useful for the design of weight management trials utilizing exercise, the potential identification of groups of participants for whom exercise may be most effective, and to identify areas for future investigation.

## Methods

This systematic review was performed and reported in accordance with the Preferred Reporting Items for Systematic Reviews and Meta-Analysis (PRISMA) guidelines [31], [32].

### Objectives

The objective of this systematic review was to address the question:

Does increased exercise or physical activity alter ad-libitum daily energy intake or macronutrient composition in healthy adults?

### Eligibility Criteria

Primary source articles published in English in peer-reviewed journals were eligible for inclusion in this systematic review if data were presented on energy and/or macronutrient intake by level of exercise or physical activity or changes in energy or macronutrient intake in response to acute exercise or exercise training. Specific eligibility criteria included: *Types of studies:* Cross-sectional, acute/short-term (exercise duration ranging from a single 30-min exercise bout to daily exercise over 14 days), and both non-randomized and randomized trials. *Types of participants:* Healthy adults (age 18–65 years). *Types of exercise interventions*: Aerobic and resistance exercise. *Types of outcome measures*: No restrictions were placed on the assessment methods for the primary outcome (energy/macronutrient intake). *Other criteria*: There were no restrictions on the length of interventions or the types of comparisons. We included cross-sectional comparisons between participants differing by level of exercise or physical activity and longitudinal pre/post within group changes vs. non-exercise control or vs. a different level of exercise. Articles were excluded if they provided no data on energy or macronutrient intake by level of exercise or physical activity, manipulated or controlled energy intake, or were conducted in non-recreational athletes or individuals with chronic disease(s).

### Information Sources

Studies were identified by searching electronic data bases, related article reference lists, and consulting with experts in the field. The search was applied to PubMed (1990-present) and adapted for Embase (1990-present). The last search was conducted on January 4, 2013. The search was developed as a collaborative effort of the research team in consultation with a Kansas University reference librarian and conducted by a co-author (SDH). No attempts were made to contact study investigators or sponsors to acquire any information missing from the published article.

### Search Strategy

We used the following search terms in PubMed and Embase to identify potential articles with abstracts for review: exercise[ti (title), ab (abstract) ] or “physical activity”[ti,ab] or “energy expenditure”[ti,ab] OR “resistance training”[ti,ab] OR “strength training”[ti,ab]) AND (diets[ti,ab] OR diet[ti,ab] OR dieting[ti,ab] or “energy intake”[ti,ab] OR “energy restriction”[ti,ab] OR “nutrient composition”[ti,ab] OR “appetite”[ti,ab]). Additional search terms were applied to eliminate case reports and studies involving participants with chronic disease, and to retrieve studies published in English and conducted in adults (age 18–65 years). Word truncation and the use of wildcards allowed for variations in spelling and word endings.

### Study Selection

Retrieved abstracts were independently assessed for eligibility for inclusion in the review by 2 investigators and coded as “yes”, “no” or “maybe.” All investigators who participated in eligibility assessments were trained regarding study inclusion/exclusion criteria and completed practice eligibility assessments on 50 test abstracts prior to actual coding. Eligibility assessments on the practice abstracts were reviewed by the primary author (JED) and any coding problems were discussed. Disagreements regarding eligibility for inclusion were resolved via development of consensus among all co-authors. Full text articles for abstracts coded as “yes” or “maybe” were retrieved and reviewed by 2 independent co-authors prior to inclusion in the review. An excel spread sheet was developed and used to track eligibility status.

### Data Collection

Extracted data was entered into the University of Kansas secure, REDCap (Research Electronic Data Capture, Version 4.14.5) data base [33]. A REDCap data extraction form was developed, pilot tested on a sample of 10 studies (at least 2 studies of each of the 4 study designs included in this review), and revised accordingly. Relevant data were extracted from each manuscript by one author and verified by a second author. Disagreements were resolved by group discussion. Data extracted from each article included basic study information (design, sample size, groups compared, exercise or physical activity groups/intervention(s), participant characteristics (age, gender, BMI, minority status), energy and macronutrient assessment method, and results.

### Risk of Bias in Individual Studies

Risk of bias for randomized trials was independently evaluated by two authors using the Cochrane risk of bias tool [34]. Risk of bias was assessed in the following domains: selection bias, performance bias, detection bias, attrition bias, reporting bias, and other bias. A third reviewer resolved any discrepancies in bias coding. Studies were not excluded on the basis of risk of bias.

### Synthesis of Results

Articles were grouped by study design: cross-sectional, acute/short-term, non-randomized, and randomized trials. Considerable heterogeneity existed within study groups for several important study parameters. These parameters included: 1) participant characteristics (age, gender, BMI), 2) physical activity assessment methods (questionnaires, pedometers, accelerometers), 3) exercise prescriptions (mode, frequency, intensity, duration), 4) comparison groups (interventions: pre vs. post-exercise, exercise vs. non-exercise control, varying amounts), 5) intervention length, and 6) energy and macronutrient assessment methods (food frequency questionnaire, weighed and un-weighed food records, direct observation weigh and measure technique). A meta-analysis was therefore considered inappropriate. Results based on the extracted data were instead synthesized and presented grouped by study design.

## Results

**Figure 1:**
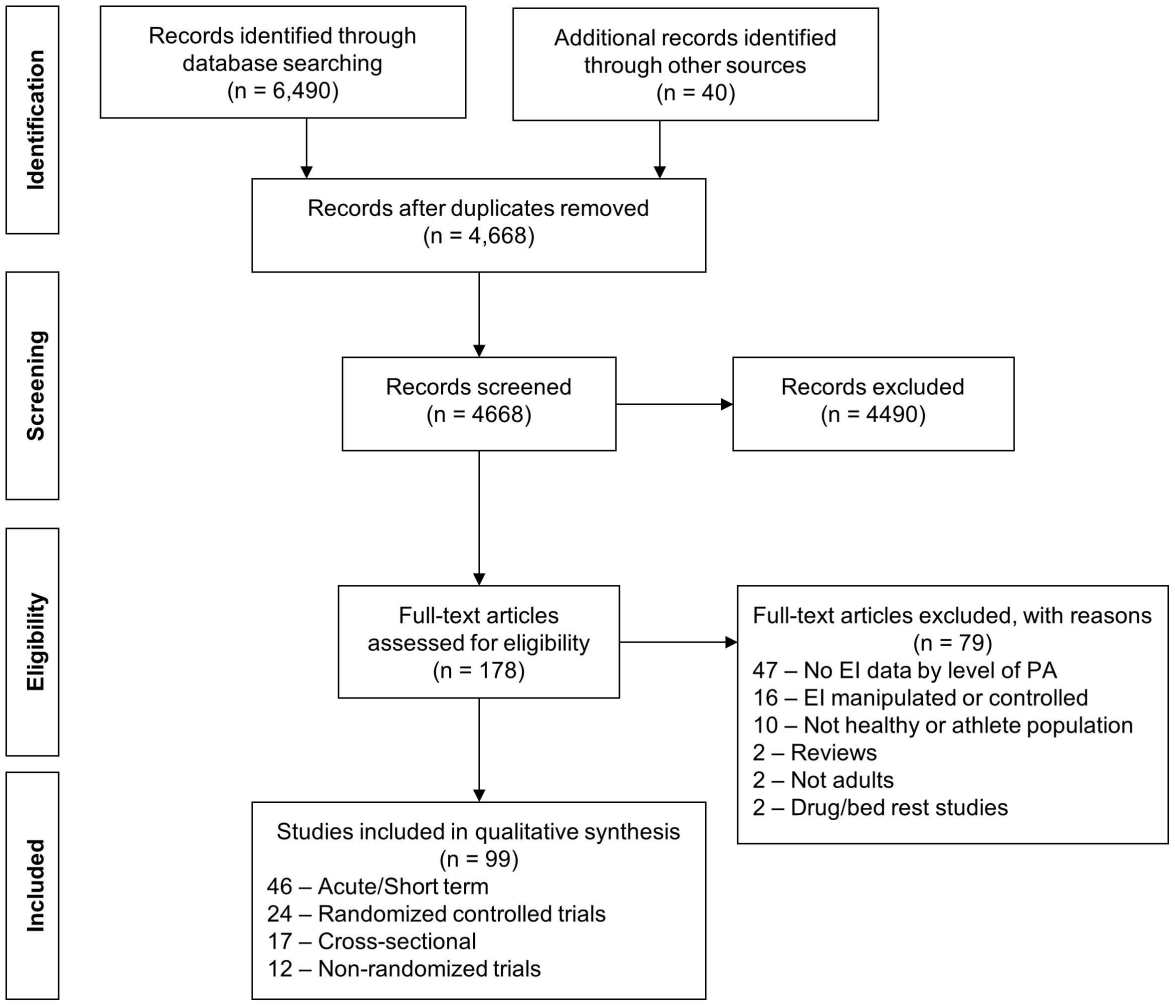
Flow diagram for identification, screening, assessing eligibility, and inclusion in systematic review.

The initial database search plus hand searching identified 4,668 unique records of which 4,490 were excluded based on review of title and abstract. Full-text articles for the remaining 178 citations were reviewed of which 79 articles did not satisfy our inclusion criteria and were excluded. Thus 99 articles representing 101 studies were included in the review (Figure 1).

### Cross-Sectional Studies

The 17 cross-sectional studies identified comprised ∼17% of the total number of studies included in this review (Table 1).

**Table 1:** Cross-sectional/Correlational Studies.

Study	Participants	Activity/Exercise Assessment	Comparison Group(s)	Dietary Assessment	Results
Butterworth *et al.* (1994) [50]	34 women: Age = 29.7 (5.5); BMI = NR; % Fat = 22.7 (6.4) from skinfolds	Portable accelerometer 1 random day each wk over a 10 wk period. VO_2_ max on a treadmill.	NA: Correlational study	10 repeated 24 hr diet records, randomly assigned, over a 10 wk period	EI per kg body weight was significantly correlated with energy expenditure. No significant correlations were found between nutrient intake/1,000 kcal and either VO_2_ max or mean 24-hour energy expenditure (kcal/day)
Camoes *et al.* (2008) [35]	Total Sample (1483 women & 921 men): Age = range 18–92; BMI = range 25.2–27.0	EPIPorto PA questionnaire: included, occupational, domestic and leisure-time activities.	Sedentary: Less than 10% of EE at a moderate or high intensity level (4 METs) during occupational activities, leisure time or throughout the day. Active: All others.	Validated semi-quantitative food-frequency questionnaire assessing dietary intake over the previous year.	There were no significant differences in daily nutrient intake by activity group for women. Active men had significantly higher daily EI and lower PRO intake as a percent of EI than sedentary men.
D'Angelo *et al.* (2010) [41]	51 women in a university required course: Age = NR; BMI = NR	International PA Questionnaire	3 groups based on percentiles: Group 1 (n = 17) = 1^st^ to 32^nd^ % ile (0 min/wk); Group 2 (n = 17) = 33^rd^ to 65^th^ % ile = 95 (35.6) min/wk; Group 3 (n = 17) = 66^th^ to 100^th^ % ile = 231.7 (85.9) min/wk	3-day food record (2 weekdays/1weekend day)	EI was significantly higher in group 3 compared to the 2 other groups.
Duvigneaud *et al.* (2007) [42]	362 women: Age = 51.4 (12.9); BMI = 24.1(3.5); 485 men: Age = 50.2 (13.3); BMI = 25.4 (3.1)	Flemish PA Computerized Questionnaire.	3 groups based on hrs/wk of sports participation: 0 (n = 129), <3.5 (n = 157), ≥3.5 (n = 199).	3-day food record ( 2 week days/1 weekend day)	No significant between group differences for energy, PRO or CHO intake. Fat intake (kcal/day and % EI) was significantly lower in the group reporting ≥3.5 hrs/wk of sport participation vs. the 0 hrs/wk group.
Eaton *et al.* (1995) [36]	Total Sample (1206 women & 798 men; Minorities = 9.3%): Age = 37.4 (5.7); BMI = 26.1 (5.7)	“At least once a week, do you engage in any regular activity such as brisk walking, jogging, bicycling, etc., long enough to work up a sweat?”	No = sedentary, Yes and did 1–3 times/wk = moderately active. Yes and did 4–7/wk = very active.	Willett Food Frequency Questionnaire	No significant difference in EI between activity groups. The more active groups had higher intakes of PRO and CHO. Total fat, saturated fat, and the percentage of kcals from fat and saturated fat were lower in the active groups.
Gilliat-Wemberly *et al.* (2001) [14]	32 women: Active (n = 18): Age = 42 (3); BMI = 21.6 (1.6) Sedentary (n = 14): Age = 42 (4); BMI = 22.8 (2.4)	Self-report	Active: Minimum of 6 hrs/wk and have maintained this level of activity for a minimum of 5 years. Sedentary: Exercise <2 hrs/wk and maintained this lifestyle for a least 5 years.	7-day food record – calibrated scales used to weigh food.	Active women had significantly lower fat intake as a % of total kcal compared to their sedentary counterparts. No between group differences in EI or in PRO, CHO, fat, alcohol, caffeine, fiber (grams/day).
Hornbuckle *et al.* (2005) [43]	69 women: Age = 51.4 (5.4); BMI = 30.9 (6.8)	7-day pedometer	<5000 steps/day; 5000–7499 steps/day; >7500 steps/day	3-day food record	No significant difference in EI between groups.
Jago *et al.* (2005) [49]	Total Sample (722 women & 469 men; Minorities = 24%): Age = 29.7 (5.1); BMI = 27.3 (6.7)	Compared to other people your age and sex, how would you rate your PA outside of work during the past year?	1 = Physically inactive; 2; 3 = Moderately active; 4; 5 = Very active	Youth/Adolescent Food Frequency Questionnaire (YAQ)	There was a significant difference in total EI across the five activity groups after controlling for BMI, age, income, ethnicity, gender, and ethnicity by gender interaction terms. Pairwise comparisons indicated that this difference was significant between groups 3 and 5. There was also a significant difference in the percentage of EI from fat by activity level with group 2 consuming a larger proportion of energy from fat than group 4. No significant between group differences in either PRO or CHO consumption.
Lake *et al.* (2009) [37]	Total Sample (29 women; 44 men): Age = 18 (1.0); BMI = NR; Convenience sample of college students studying sport (24 men, 8 women) or non- sport (20 men, 21 women) subjects.	PA component of the ‘Youth NEWS’ (Neighbourhood Environment Walkability Survey)	Sport vs. non-sport students and categories of time spent in sedentary pursuits (homework, TV, DVD etc.)	The European Prospective Investigation of Cancer food frequency questionnaire. Assessed dietary intake over the past year.	Sport students had a significantly lower intake of fat and a significantly higher percentage energy from protein compared to the non-sports students. Sedentary behaviors were significantly associated with less healthy eating patterns. Higher total EI, higher fat percentage energy from fat and lower CHO intakes were significantly associated with more time spent watching DVDs on weekends.
Lee *et al.* (2010) [44]	39,876 women (Minorities = ∼5%) Age by activity group: <7.5 MET-hrs/wk = 54.1 (6.8); 7.5<21 = 54.3 (6.9); ≥21 = 54.2 (6.9). BMI by activity group: <7.5 MET-hrs/wk = 26.9 (5.5); 7.5 to <21 = 25.5 (4.5); ≥21 = 24.5 (4.1)	College Alumni Health Study PA Questionnaire.	3 Groups: 1) <7.5 MET-hrs/wk equivalent to <150 min/wk of moderate- intensity PA); 2) 7.5 to <21 MET-hrs./wk.; 3) ≥21 MET-hrs/wk.(equivalent to ≥420 min/wk moderate-intensity activity.	Willett Food Frequency Questionnaire.	No significant between group differences for EI, fat intake, or servings of fruits and vegetables by activity group.
Lissner *et al.* (1997) [45]	361 women: Age: Sedentary = 50.4 (6.3); Somewhat Active = 48.1 (6.4); More Active = 48.9 (5.1); BMI: Sedentary = 25.7 (5.3); Somewhat Active = 24.3 (3.9); More active = 23.2 (3.3)	Gothenburg PA Questionnaire	3 Groups: Sedentary = almost inactive; Somewhat active = engaged in at least 4 hrs. of activity/wk; More active = greater than 4 hrs/wk.	Food frequency questionnaire	No significant between group differences for EI or fat intake (g/day and % total EI)
Matthews *et al.* (1997) [47]	Total Sample (494 women, 425 men); Age: Women = 48.4 (10.6); Men = 49.8 (10.2). BMI: Women = 28.1 (5.9); Men = 27.8 (4.4)	Questionnaire: “Do you exercise on a regular basis?” and if yes asked about frequency (times/wk), duration (min/session) and mode. If no answer assumed they engaged in no explicit leisure time PA.	Inactive = 0 to 29 min/wk.; Active = 30 min or more/wk; Active sub-categories:>30 to 60 min/wk; 61 to 120 min/wk; ≥121 min/wk	7-day or 24-hour diet recalls.	No significant difference in EI or macronutrient intake between the 4 activity groups.
Mulligan *et al.* (1990) [46]	21 women: Non-runners (n = 5); Moderately active runners (n = 9); Very active runners (n = 7); Age by activity group: Non-runners = 30.6 (5.6); Moderately active runners = 37.2 (4.2); Very active runners = 29.8 (8.0)	Activity diary over 2 months and VO_2_ max.	Non-runners: distance/wk. = 0; VO_2_ max = 41.7 (8) ml/kg/min; Moderately active runners: 39.5 miles/wk.; VO_2_ max = 39.5 (10.1) ml/kg/min; Very active runners: 54.4 miles/wk.; VO_2_ max = 66.8 (7.5) ml/kg/min	Weigh and measure diet record collected over 24 hrs on 3 random consecutive days each wk over 2 months.	No significant between group differences in EI.
Perry *et al.* (1992) [48]	70 women: Distance runners (n = 19); Recreational joggers (n = 19); Aerobic dancers (n = 17); Inactive controls (n = 15)). Age: Runners = 37.2 (9.0); Joggers = 32.6 (8.3); Aerobics = 32.2 (6.0); Controls = 33.3 (7.3). BMI: Runners = 21.7 (NR); Joggers = 20.4 (NR); Aerobics = 20.8 (NR); Controls = 22.3 (NR)	Recruited by activity level.	Long distance runners: Averaged at least 26 mi/wk, mean = 40 mi/wk; Recreational joggers: Averaged at least 6 mi/wk., mean = 16 mi/wk.; Aerobic dancers: Taught at least 6 aerobic dance classes/wk, mean = 9.4 hrs/wk.; Inactive controls: Exercised less than one time/wk and not on a regular basis.	One-day food record	EI was not significantly difference between groups.
Rintala *et al.* (2010) [38]	16 twin pairs (7 monozygous, 9 dizygous; 11 male and 5 female pairs): Age = 60 (range 50–74); BMI: Inactive = 26.7 (3.5); Active = 21.5 (6.4)	Questionnaire: 3 structured questions; mean duration of one activity session with five response alternatives, monthly frequency of activity with six response alternatives and activity intensity based on the following question: “Is your PA during leisure time about as strenuous on average as: (i) walking, (ii) alternately walking and jogging, (iii) jogging, (iv) running?”	Active/inactive co-twins discordant for PA over a 32 year period. Inactive co-twins averaged 8.8 MET-hrs/day less activity than their active co-twin.	Food diary: 3 week days and 2 weekend days	No significant difference in absolute EI (kcal/day) between active and inactive co-twin. REI (kcal/kg) was significantly higher in active vs. inactive co-twins. No significant difference between active and inactive co-twins for either absolute (g/d) or relative intake (%EI) of PRO, CHO or fat.
Van Pelt *et al.* (2001) [40]	137 men: Young sedentary (YS): n = 32; Old sedentary (OS): n = 34; Young active (YA) : n = 39; Old active (OA): n = 32. Age: YS = 26 (1); OS = 62 (1); YA = 27 (1); OA = 63 (1). BMI: YS = 24.2 (0.7); OS = 26.7 (0.7); YA = 22.7 (0.4); OA = 23.5 (0.3)	Recruited by activity level.	Active: Local runners and triathletes who placed in the top 25% of their age divisions in either 10-km running events or triathlon competitions. Sedentary: Performed no regular PA.	Food diary over 4 consecutive days (3 weekdays and 1 weekend day)	EI (kcal/day) was significantly higher in the YA vs. the YS but did not differ by activity level in older men. No significant between group difference for macronutrient intake (%fat, CHO or PRO) in either the young or old group.
Van Wallenghen *et al.* (2007) [39]	Total Sample (15 women, 14 men): Age: Sedentary = 26.0 (1.0); Active = 23.0 (1.0). BMI: Sedentary = 23.5 (0.8); Active = 23.1 (0.7)	Habitual PA including both planned exercise (jogging, cycling, etc.) and daily living-type activities (housework, yard work, etc.) was determined by self-reported time (min/wk) spent participating in moderate and vigorous PA.	Active = active subjects spent ≥150 min/wk engaged in moderate and/or vigorous PA for ≥2 years.	Self-reported 4-day food intake record.	EI (kJ/day) was significantly higher in the active group. The % energy from fat was lower in the active group, % energy from CHO was higher in the active group. No between group differences in % energy from PRO.

*Note*, Values are means (standard deviation) unless otherwise noted. Abbreviations: PA = Physical Activity; CHO = carbohydrate; EE = energy expenditure; EI = energy intake; NR = not reported; NA = not applicable; PRO = protein; REI = relative energy intake; MET = Metabolic equivalent of task; kcal = kilocalories; KJ = kilojoules; hrs = hours; min = minutes; wk = week.

### Cross-Sectional Studies: Study Characteristics

#### Sample size

Median (range) sample size for studies that included both men and women was 447 (14–921) for men and 428 (10–1483) for women. In studies that included only men or women the median sample size for men was 137 (one study) and women was 60 (21–38,876).

#### Comparisons

Seven studies compared 2 groups (active vs. sedentary) [14], [35]–[40]. Six studies compared 3 groups [41]–[46] while 3 studies compared physical activity over 4 or more groups [47]–[49]. One study reported the correlation between physical activity level and energy intake [50].

#### Physical activity assessment

Seven studies (∼41%) employed a physical activity questionnaire [14], [35], [37], [41], [42], [44], [45], 5 studies (∼29%) used a single item self-report or self-report physical activity rating scale [36], [38], [39], [47], [49], and one study used a physical activity diary[46]. Two studies used objective assessments of physical activity (accelerometers/pedometers) [43], [50] while 2 studies recruited participants based on their self-reported participation in recreational aerobic sports [40], [48].

#### Energy/Macronutrient intake assessment

Food frequency questionnaires were used in 6 studies [35]–[37], [44], [45], [49] while 4 studies used non-weighed 3-day food records [38], [41]–[43]. One [48], 4 [39], [40] and 7-day [47] non-weighed food records, 3 [46] and 7-day weigh and measure food records [14] and repeated 24-hour recalls were also employed in 2 studies [47], [50].

### Cross-Sectional Studies: Participant Characteristics

#### Age

The median (range) age across the 15 studies that reported age was 42 (18–60) years.

#### Gender

Nine studies included both men and women [35]–[39], [42], [47], [49], 8 studies included women only [14], [41], [43]–[46], [48], [50] while one study included only men [40].

#### BMI

The median (range) BMI across 13 studies that reported BMI was 24.8 (21.3–30.9) kg/m^2^.

#### Minority representation

In the 3 of 17 studies (18%) that provided data, minority representation ranged from 5% [44] to 24% [49] of the total sample.

### Cross-Sectional Studies: Results

#### Energy intake

Seven of 17 (41%) cross-sectional studies indicated significantly higher absolute (kcal/day) [35], [39]–[41], [49] or relative (kcal/kg/day) energy intake [38], [50] in active compared with less active groups. There were no apparent differences in either basic study design parameters including sample size, assessment methods for both energy intake and physical activity, the number and type of comparison groups, or participant characteristics such as age, gender or BMI between studies that did and did not report a significant association between physical activity and energy intake.

#### Macronutrient intake

Thirteen of 17 studies presented data on the intake of one or more macronutrients (fat, carbohydrate, protein). All 13 studies included data on fat intake. Six studies (46%) reported either lower absolute (grams) [36], [42] or relative fat intake (% total energy intake) [14], [36], [37], [39], [42], [49] in groups with higher levels of physical activity while 7 studies (54%) that reported fat intake by activity level failed to find a significant association [35], [38], [40], [44], [45], [47], [50]. Eleven studies provided data on both carbohydrate and protein intake. Two studies (18%) reported higher carbohydrate intake in active groups [36], [39], while 9 studies (82%) found no association between the level of physical activity and carbohydrate intake [14], [35], [37], [38], [40], [42], [47], [49], [50]. Relative protein intake was higher (2 studies) [36], [37], lower (one study) [35] or not different (8 studies) [14], [38]–[40], [42], [47], [49], [50] between groups reporting higher vs. lower levels of physical activity.

### Acute Studies

The 40 acute studies comprised ∼40% of the total studies identified for this review (Table 2). All acute studies employed cross-over designs, which compared energy intake assessed over a time frame of 24 hours or less following an acute exercise bout.

**Table 2:** Acute and Short-Term Studies.

Study	Participants	Intervention	Meals	Results: Exercise	Results: Diet
Balaguera-Cortez *et al.* (2011) [77]	10 men: Age = 21.3 (1.4); BMI = 23.7 (2); VO_2_ max = 58.1 (7.3) ml/kg/min	45 min aerobic: Treadmill at 70% of VO_2_ peak; 45 min RT: 3 sets of 12 reps of 8 different exercises; Control: 45 min rest; experiment started at 7 AM, 45 min exercise, 30 min rest, followed by assessment of EI.	Buffet-type breakfast, foods and drinks for 30 min and instructed to eat ad libitum and to satiation.	Total weight lifted = 14,432 (3,551) kg. Aerobic exercise was at 71 (7%) VO_2_ peak, and EEEx was 2818 (418) kJ for the 45 min. Estimated EEEX from the RT trial was 1,350 kJ for 45 min. Control EE was 256 kJ for 45 min.	No significant effect of trial on total EI, or CHO, fat, or protein both in grams and as a percent of total EI. Also no differences between trials in solid food or drink consumption as a % of total EI.
Deighton *et al.* (2012) [81]	12 men: Age = 23.0 (3.0); BMI = 22.9 (2.1); VO_2_ max = 57.9 (9.7) ml/kg/min	Control: 60 min rest; Exercise: 60 min treadmill run at 70% max VO_2_ performed either in a fasted (8 AM) or fed (12 PM) state.	Standardized breakfast served at 9:30 AM. Buffet meal offered at 1:30 and 5:30 PM.	Net EEEx fasted trial = 3247 (423 kJ); Net EEEx fed trial = 3234 (435) kJ	No significant differences in EI between trials resulting in a negative energy balance in the exercise trials relative to control after accounting for differences in EEEx.
Erdman *et al.* (2007) [64] Intensity trial	5 women, 2 men: Age = 24.4 (0.6); BMI = 21.4 (0.8); Activity level/fitness = NR	Low intensity exercise: 30 min at 50 W (60 rpm). Higher intensity exercise: 30 min at 100 W (60 rpm). Control: 45 min rest.	Sandwiches consisting of bread, butter and ham (2.73 kcal/g, energy percent: 44.4% carbohydrate, 16.2% protein and 39.4% fat). Test meal offered 15 min post exercise	EEEX low intensity = 85.6 Kcal; EEEx higher intensity = 171.2 Kcal	Food (g) and EI (kcal) were not different after either intensity compared to the control.
Erdman *et al.* (2007) [64] Duration trial	3 women, 4 men: Age = 24.8 (0.7); BMI = 22.1 (NR); Activity level/fitness = NR	Cycle ergometer exercise (60 rpm) for 30, 60, 120 min at 50 W.	Same as above	EEEx 30 min = 85.6 Kcal; EEEx 60 min = 171.2 Kcal; EEEx 120 min = 342.4 Kcal	EI and food intake after the 30 and 60 min exercise bouts was not different from controls. EI and food intake following the 120 min bout were significantly greater than control.
Farah *et al.* (2010) [96]	10 men: Age = 35 (6); BMI = 28.2 (2.4); VO_2_ max = 43.0(6.4) ml/kg/min	3–4 day trials: Control: No exercise on days 1–3. EX-1: One exercise session on day 3; treadmill walking, 50% VO_2_ max. EX-3: exercise sessions on each day 1–3 at 50% VO2max.	Reported to metabolic suite on the morning of day 4. Presented with ad lib buffet style breakfast and lunch.	Net EEEx = 2.98 (0.11) MJ in EX-1 and 8.92 (0.30) MJ in EX-3.	There were no differences between trials in energy, fat, carbohydrate, or protein intakes at breakfast, but energy, carbohydrate, and protein intakes at lunch were significantly higher in EX-3 than in both CON and EX-1
Finlayson *et al.* (2009) [69]	24 women: Age = 24.0 (6.1); BMI = 22.3 (2.9); Activity level/fitness = NR	Control: 50 min sitting. Exercise: 50 min cycle ergometer at 70% of maximum HR.	Test meal offered 40 min post trial.	Estimated EEEx = 189.3 (13) kcal.	EI was not different between exercise and control conditions.
George & Morganstein (2003) [73]	24 sedentary women (12 normal and 12 overweight): Age = 35 (8); BMI = 22 (1) Normal weight; 28(1) overweight	Exercise = walking on a treadmill at 60% of max HR for 60 min. Non-exercise = sedentary activities for 60 min.	Ad libitum lunch at the university cafeteria. Test meal offered approximately 30 min post trial	Estimated EEEx = 150–200 kcal depending on body weight.	EI was not different between exercise and control in either normal or overweight women.
Harris *et al.* (2008) [61]	80 sedentary men: Age = 30 (8); BMI = range 22–29; 5 groups: 1) normal weight, low-restraint, non-dieting; 2) normal weight, high-restraint, non-dieting; 3) overweight, low-restraint, non-dieting; 4) overweight, high-restraint, non-dieting; and 5) overweight, high-restraint, dieting.	Control: 60 min rest. Exercise: 60 min treadmill exercise at 60–65% age-predicted max HR.	Ad libitum lunch at the university cafeteria. Other EI over 12 hrs post exercise was assessed by diet recall conducted by phone. Test meal offered approximately 15 min post trial.	EEEx = NR	Exercise had no significant impact on post exercise EI in any study group.
Imbeault *et al.* (1997) [51]	11 men: Age = 24.4 (3.3); BMI = 23.3 (2.3); VO_2_ max = 56.7(5.0) ml/kg/min	Control: seated for the same duration as the low intensity exercise condition. Low intensity exercise: Treadmill walking at 35% VO_2_ max, duration to reach EEEx of ∼2050 kJ. High intensity exercise: Treadmill running at 75% VO_2_ max to reach EEEx of ∼2050 kJ.	Buffet meal 15 min post trial.	EEEx low intensity = 2054 (45) kJ. EEEx high intensity = 2022 (38) kJ.	There were no significant between group differences for absolute EI. Relative EI (EI minus net EEEx) was significantly lower for high intensity exercise than both control and low intensity exercise.
Jokisch *et al.* (2012) [78]	20 men; 10 active (exercise ≥150 min/wk); 10 inactive (exercise≤60 min/wk): Age = 21.2 (1.9); BMI = 23.4 (1.7)	Control: 45-min reading. Exercise; 45 min cycle ergometer at 65–75% of age predicted max HR to elicit EEEx of approximately 450 Kcal.	Ad libitum buffet lunch. Test meal offered 60 min post trial.	EEEx active = 456 (9) kcal/45 min. EEEx inactive = 451 (12) kcal/45 min both estimate using American College of Sports Medicine equations for energy expenditure during leg cycling.	EI was significantly lower at the ad libitum meal following exercise as compared to control in the inactive group. There was no effect of exercise on EI in the active group. No difference in meal macronutrient intake was found.
Keim *et al.* (1990) [90]	12 sedentary to moderately active women: Age = 30 (1); BMI = 27.7 (NR)	4 days acclimatization to exercise and 14 days at the specified exercise level. 3 conditions: No exercise (NO-EX): subjects maintained usual daily activity. Moderate duration exercise (M-EX): achieve an energy expenditure 12.5% above baseline. Long duration exercise (L-EX): achieve an energy expenditure 25% above baseline.	Participants were free-living. Required to report to a metabolic kitchen daily to consume breakfast and dinner, to pick up lunch and snacks, and to perform prescribed exercise programs.	EEEx goals for 12.5% and 25% above baseline were achieved with exercise at 72 (1)% of predicted VO_2_ max for both exercise groups. Duration of exercise ranged from 31–49 min in the M-Ex group and 51–88 min in the L-EX group.	There were non-significant increases in EI in both the M-EX (5%) and L-EX (8%) groups. Daily intake of carbohydrates, protein or fat averaged over the study period was not affected by exercise.
Keim *et al.* (1996) [91]	15 weight reduced obese women, Aerobic exercise (n = 8): Age = 31 (2 SEM); BMI = 28.1 (1.6 SEM); Resistance exercise (n = 7):Age = 31 (3 SEM); BMI = 29.3 (2.0 SEM); Activity level/fitness = NR	14 days Aerobic exercise: Supervised treadmill exercise 5 days/wk, 75% VO_2_ max, EEEx at 29% of RMR. Resistance exercise: Supervised, 1 set of 8 reps at 50–60% 1-RM.	All meals provided in a metabolic ward.	EEEx aerobic exercise = 354 kcal/session. EEEx resistance exercise = 96 kcal/session.	EI during the intervention was not significantly different from a 3 wk. control period in either the aerobic or resistance training groups. EI for both the aerobic and resistance training groups were not significantly different during the intervention period despite the higher levels of EEEx in the aerobic exercise group.
Kelly *et al.* (2012) [79] Normal hydration condition	10 men: Age = 21.4 (1.3); BMI = 23.9 (2.1) Active	Control: 45 min rest. Exercise: 45 min treadmill running at 70% VO_2_ peak.	Buffet breakfast offered 30 min post trial.	EEEx = 2949 (207) kJ.	There was no significant difference in EI or macronutrient composition between trials. Relative EI (post-exercise EI minus (EEEx+EPOC) was significantly higher in control vs. the exercise trial.
King JA *et al.* (2010) [58]	14 men: Age = 21.9 (0.5 SEM); BMI = 23.4 (0.6 SEM); VO_2_ max = 55.9 (1.8) ml/kg/min	Control: 7 hrs rest. Exercise: 60 min subjectively paced walk on a level motorized treadmill.	Ad libitum buffet foods that were identical for each meal. Test meal offered 30 min and 4 hrs post trial.	Walks were completed at a mean of 42.5(2)% of VO_2_ max and generated a net EEEx (exercise minus rest) of 2008 (134) kJ.	EI was not significantly different between the control and brisk walking trials. After adjusting for the energy expenditure of walking, there was an energy deficit of 1836 (130) kJ in the brisk walking trial compared with the control trial. No significant between trial differences absolute energy intake from fat, CHO or PRO.
King JA *et al.* (2010) [57]	9 men: Age = 22.2 (0.8); BMI = 23.6 (0.4); VO_2_ max = 60.5 (1.5) ml/kg/min	Control: 8.5 hrs rest. Exercise: 90 min treadmill run at 68.8 (0.8)% of max VO_2_ followed by 8.5 hrs rest.	Ad libitum cold buffet offered at 2.5 and 9 hrs. Hot meal at 5.5 hrs.	Net energy expenditure (exercise minus rest) was EEEx = 5324 (186) kJ or 1273 (45) kcal.	No effect of trial on EI or macronutrient intake. After accounting for EEEx participants remained in energy deficit in the exercise as compared to the control trial.
King JA *et al.* (2011) [82]	12 men: Age = 23.4 (1); BMI = 22.8 (0.4); VO_2_ max = 57.3 (1.2) ml/kg/min	Control: 7.5 hrs rest. Exercise: 90 min treadmill run at 70% VO2 max.	Test meal at 2 and 4.75 hrs. Buffet meal at 8 hrs.	Net EEEx (exercise minus rest) = 4715 (113) kJ.	No significant difference in EI or macronutrient intake between exercise and control.
King JA *et al.* (2011) [56]	14 active men: Age = 22 (0.5); BMI = 23.2 (0.6)	Control: 7.5 hrs rest. Exercise: Swimming – 6 ten min blocks at a moderate intensity (perceived exertion between 12 and 14).	Ad libitum buffet meal offered at 3 hrs (∼12:00) and 7.5 hrs (∼16:30) into the trial.	Net estimated EEEx (exercise minus resting)+1921 (83) kJ.	No significant difference in EI or macronutrient intake between swimming and control trials.
King NA *et a*l. (1997) [94]	8 active men: Age = 26.0 (5.2); BMI = 22.4 (1.8)	Each condition was performed over 2 consecutive days. Control: No exercise (R1 and R2). Exercise (Ex1 and Ex 2): 2 session in one day of treadmill running at ∼70% max HR for 50 min (100 min at 70% max HR)	Food and drink intake were monitored using a set of digital weighing scales and a self-record diary.	The combined EEEx for both Ex sessions was 1200 kcal.	No significant differences in EI were noted between R1 and R2, Ex1 and Ex2 or Ex1 and R1 or Ex2 and R2. Also no differences between trials for PRO, FAT or CHO as a % of kcal intake.
King NA *et al.* (1996) [74]	13 women: Age = 22.6 (2.3); BMI = 21.9 (1.6); VO_2_ max = 37.0 (3.0) ml/kg/min	Control: 50 min rest. Exercise: cycle ergometer, 50 min at 70% VO_2_ max.	Ad libitum lunch containing either 7 high-fat foods or 7 low-fat foods. Test meal offered 15 min post trial.	EEEx was approximately 350 kcal at 73% VO_2_ max.	EI after exercise increased slightly (9%), however was not significant when compared to the rest condition. EI was increased when participants consumed the high-fat lunch as compared to the low-fat lunch for both the rest and exercise conditions.
King NA *et al.* (1995) [59]	24 men; assigned to either a treadmill running (n = 12) or cycle ergometer (n = 12) study. Age = Run: 21.4 (3.4); Cycle: 24.4 (5.3); BMI = Run: 22.9 (NR); Cycle: 22.8 (NR); VO_2_ max Cycle = 49.3 (2.2) ml/kg/min; VO_2_ max Run = 47.9 (8.2)	4 test conditions for both the run and cycle groups. Control, high fat/low carb lunch options. Control, low fat/high carb lunch options. Exercise (70% VO_2_ max), high fat/low carb lunch options. Exercise (70% VO_2_ max), low fat/high carb lunch options.	Ad libitum test lunch. Ad libitum food provided for consumption on the rest of the day assessed by records. Test meal offered immediately post trial.	EEEx for both cycling and running was approximately 600 kcal over 45 min.	No significant main effect of exercise on EI; however, EI was higher when high fat/low carb food options were available. There was a significant main effect of exercise on relative energy intake (REI: test meal intake minus EEEX). REI was significantly suppressed in the low fat/high carb treatment but not the high fat/low carb treatment vs. control.
King NA *et al.* (1994) [60] Intensity trial	11 active men: Age = (range 21–27); BMI = 24.2 (NR)	Control: Seated rest for ∼45 min. High intensity exercise: Cycle at 70% VO_2_ max ∼30 min (mean = 27 min). Low Intensity Exercise: Cycle at 30% of VO_2_max for ∼60 min (mean = 63 min).	Ad libitum test meal offered 15 min following completion of the trial. EI over the rest of the experimental day was assessed by food records.	EEEx high intensity = 340 (27.6) Kcals. EEEx low intensity = 359 (41.5) Kcals.	No significant difference in absolute EI or macronutrient intake at the test meal between the 3 treatments. Food diary data showed no significant difference between the 3 treatments for the remainder of the day.
King NA *et al.* (1994) [60] Duration trial	12 active men: Age = range (22–31); BMI = 23.2 (2.2)	Control: Seated rest for ∼45 min. Short duration exercise: 26 min at 70% VO_2_ max. Long duration exercise: 52 min at 70% VO_2_ max.	Same as above	EEx short duration = 296 (38.4) kcal. EEEx long duration = 541 (52.2) kcal.	No significant difference in absolute EI or macronutrient intake at the test meal between the 3 treatments. Food diary data showed no significant difference between the 3 treatments for the remainder of the day.
Kissileff *et al.* (1990) [75] Non-Obese Participants	9 sedentary women: Age = 22.7 (4.9); BMI = 21.1 (1.8)	Control: 40 min rest. Strenuous exercise: 40 min at 90 watts on a cycle ergometer. Moderate exercise: 40 min at 30 watts on a cycle ergometer.	Test meal offered 15 min post trial.	EEEx strenuous exercise = 246.8 (24.3) Kcal. EEEx moderate exercise = 113.7 (36.7) kcal.	EI was significantly reduced compared to control following strenuous but not moderate exercise.
Kissileff *et al.* (1990) [75] Obese Participants	9 sedentary women: Age = 24.3 (4.9); BMI = 27.7 (0.9)	Same as above	Same as above	EEEx strenuous exercise = 237.2 (25.1) kcal. EEEx moderate exercise = 143.2 (33.3)	EI was not significantly different across conditions.
Laan *et al.* (2010) [65]	10 men, 9 women: Age = 22.3 (2.5); BMI = 22.5 (1.8); VO_2_ max = 60.1 (22.5) ml/kg/min	Control: 35 min rest. Aerobic exercise: 35 min cycling at 70% HRR. Resistance exercise: 5 exercises, 3 sets at 70% 1 RM, 2 sets of 10 repetitions and a 3^rd^ set to voluntary fatigue or 12 repetitions.	Test meal (large bowl of pasta salad) offered 30 min post trial.	Estimated EEEx = 290 (7) kcal for aerobic and 80 (2) kcal for resistance exercise.	EI was higher in both aerobic and resistance exercise trials compared with control. Relative EI (EI – EEEx) was lower in aerobic exercise compared with the resistance and control trials.
Larson-Meyer *et al.* (2012) [76] Runner sample	9 women: Age = 23.7 (2.4); BMI = 19.8 (1.0); VO_2_ max = 49.7 (3.0) ml/kg/min	Control: 3 hrs rest. Exercise: 60 min treadmill run at 70% VO_2_ max.	Test meal offered 2 hrs post trial.	EEEx = 483.1 (49.7) kcal.	EI and macronutrient intake were not significantly different between exercise and control trials. REI was significantly lower following exercise vs. control.
Larson-Meyer *et al.* (2012) [76] Walker sample	10 women: Age = 24.6 (6.9); BMI = 21.1 (3.4); VO_2_ max = 33.9 (3.7) ml/kg/min	Control: 3 hrs rest. Exercise: 60 min treadmill walk at 70% VO_2_ max.	Same as above	EEEx = 324.6 (138.1) Kcal.	EI was not significantly different in exercise vs. control trials. PRO and fat intake was significantly higher following exercise vs. control.
Lluch *et al.* (1998) [71]	12 women: Age = 21.7 (2.2); BMI = 22.6 (1.9); VO_2_ max = 41.0 (4.4) ml/kg/min	Rest- low fat: 50 min rest - low fat test meal. Rest-high fat: 50 min rest - high fat test meal. Exercise-low fat: 50 min cycle at 70% VO_2_ max-low fat test meal. Exercise-high fat: 50 min cycle at 70% VO_2_ max-low fat test meal.	Test meal (lunch) offered 20 min post trial.	EEEx exercise-low fat trial = 425 (60) kcal. EEEx exercise-high fat trial = 422 (59) kcal.	Neither EI at the test meal nor EI during the rest of the day were significantly different between the exercise and rest conditions for either diet. When controlling for EEEx, exercise decreased relative EI by 43%. Therefore there was no energy compensation in response to exercise during the following lunch test meal.
Lluch *et al.* (2000) [70]	13 women (unrestrained eaters: URE): Age = 22.6 (2.3); BMI = 21.9 (1.6); VO_2_ max = 37.0 (3.3) ml/kg/min. 12 women (restrained eaters: RE): Age = 21.7 (2.2); BMI = 22.6 (1.9); VO_2_ max = 41.0 (4.4) ml/kg/min	Same as above	Test meal (lunch) was offered immediately post trial.	EEEx unrestrained eaters = 1455 (213) kJ. EEEx restrained eaters = 1772 (242) kJ.	There was a significant main effect of lunch type on EI with EI increased in both high fat conditions compared with low fat. The exercise by group interaction on EI was not significant: URE increased EI after exercise whereas in RE the EI tended to decrease. When exercise induced EE was controlled for, there was a significant effect of lunch type, exercise and exercise by group interaction on relative EI: exercise decreased relative EI when compared to rest and this difference was higher in restrained compared to unrestrained eaters.
Maraki *et al.* (2005) [63]	12 sedentary women: Age = 28 (6.4); BMI = 21.3 (1.6)	Control: 60 min rest. Exercise: 60 min aerobic and muscle conditioning exercise classes. Each trial was done both in the morning (0815 to 0915) nd in the evening (1915 to 2015).	Diet was assessed on each trial day by 24-hr diet record.	Estimated EEEx exercise trial = 1233 (106.8) kJ. Estimated EEEx control trial = 234 (20.3) kJ.	No significant difference between the 4 trials in daily EI.
Martins *et al.* (2007) [68]	6 sedentary to active women, 6 sedentary to active men: Age = 25.9 (4.6); BMI = 22.0 (3.2)	Control: 60 min rest. Exercise: 60 min intermittent cycle ergometer exercise at 65% of each subject's estimated max HR (2 min warming up+17 min exercise+3 min break+17 min exercise+3 min break+17 min exercise+2 min cooling down)	Buffet test meal offered 1-hr post trial.	Estimated EE control session = 197 (37) kcal. Estimated EEEx = 492 (92) kcal.	Absolute EI at the buffet meal was significantly higher in the exercise vs, the control trial. No significant differences were observed in the percentage of energy provided by either PRO, fat or CHO. REI was significantly lower following the exercise vs with control.
Melby *et al.* (2002) [54] Non-carbohydrate supplement group	13 women: Age = 23 (0.8); BMI = 21.6 (0.2); VO_2_ max = 39.6 (0.9) ml/kg/min	Control: ∼76 min rest. Exercise: Cycle ergometer exercise at 65% VO_2_ peak to produce a net energy cost of ∼500 kcal.	Buffet meal offered 90 min post trial.	Net EEEx = 517 (6) kcal.	No significant differences between control and exercise trials in EI or macronutrient intake either at the test meal or food consumed over the remainder of the day.
O'Donoghue *et al.* (2010) [55]	9 men: Age = 20.3; BMI = 22.4 (1.6); VO_2_ max = 58.8 (5.6) ml/kg/min	Control: 45 min rest. Exercise: 45 min treadmill running at 75% VO_2_ peak. Exercise was performed at 7 AM or 5 PM.	Buffet breakfast, lunch and dinner were offered on trial days.	EEEx AM exercise = 2,831 (519) kJ.EEEx PM exercise = 2898 (570) kJ.	No significant differences between trials in absolute EI or macronutrient intake. REI was significantly lower post exercise compared with control; i.e., REI during the breakfast was significantly lower after AM exercise than either PM exercise or control. REI during the evening period was significantly lower after PM exercise than for the control trial and tended to be lower than the AM exercise trial. However, over the total 26- hr period REI was similar between all trials.
Pomerleau *et al.* (2004) [52]	13 women: Age = 22.2 (2); BMI = 22.2 (4); VO_2_ max = 44.0 (4.7) ml/kg/min	Control: rest 75 min. Low intensity exercise = treadmill walking at 40% VO_2_ peak. High intensity exercise = treadmill walking at 70% VO_2_ peak. Duration to achieve EEEx of ∼350 kcal at both exercise intensities.	Buffet lunch offered 1-hr post trial. Afternoon snacks were provided. Participants returned at 5:30 PM for a dinner buffet-type meal and left with a second bag of snacks for the evening.	EEEx low intensity exercise = 350.6 (11.1) kcal. EEEx high intensity exercise = 358.6 (10.3 kcal.	EI at lunchtime was significantly increased following high but not low intensity exercise compared to control. REI at lunchtime for both the high and low intensity exercise sessions was significantly lower than control. No significant between trial differences in total daily absolute or REI.
Stubbs *et al.* (2002) [89]	6 men: Age = 31 (5); BMI = 23.3 (2.4); VO_2_ max = 39.5 (1.5 SEM) ml/kg/min	9-day protocol with 3 conditions: Control: no additional exercise. Moderate exercise = 2×40 min sessions/day at 21.4 kJ/kg/day, cycle ergometer. High exercise = 3×40 min exercise session/day at 42.8 kJ/kg/day, cycle ergometer. Exercise performed on days 3–9.	On days 1–2 of the study subjects consumed a mandatory maintenance diet (estimated at 1.6×RMR). Throughout the subsequent 7 days they had ad libitum access to their normal daily diet. They weighed all food items and fluids on Portable Electronic Tape Recording Automated scales.	Total daily EE: Control = 11.7 MJ/day. Moderate exercise = 12.9 MJ/day. High exercise = 16.8 MJ/day.	There were no significant differences in total daily food, EI or macronutrient composition of the diet between treatments.
Stubbs *et al.* (2002) [87]	6 women: Age = 23.0 (0.6); BMI = 21.4 (1.0); VO_2_ max = 33.4 (2.5) ml/kg/min	Same as above	Same as above	Total daily EE: EE control = 9.2 MJ/day. EEEx moderate exercise = 11.0 MJ/day. EEEx high exercise = 12.1 MJ/day.	EI was significantly higher in the high exercise treatment vs. control, but not different from the moderate exercise treatment. No significant between group differences for energy from protein; however, energy from both fat and CHO were significantly higher for the high exercise treatment vs. control.
Stubbs *et al.* (2004) [92]	6 men: Age = 23 (2.3); BMI = 22.2 (2.4); Activity level/fitness = NR	7-day whole room calorimeter protocol with 2 conditions: Sedentary: 2×40 min cycle ergometer sessions to increase daily energy expenditure to 1.4×RMR. Active: 3×40 min cycle ergometer sessions to increase daily energy expenditure to 1.8×RMR.	All meals provided. Food items were weighed before and after each meal.	Sedentary daily EE = 9.75 MJ. Active daily EE = 12.77 MJ.	No significant differences in EI or macronutrient intake between treatments.
Schneider *et al.* (2009) [66]	43 sedentary women, 22 sedentary men: Age = 34.4 (10.8); BMI = 33.5 (5.5)	Control: 3 min sitting. Active: 3 min step test, up and down a 17.5 cm bench at 92 steps/min.	Participants were offered two 800 kcal portions of cookies and chips (1600 kcal total) 10 min following the trial.	EEEx = NR	No significant differences in EI between conditions.
Shorten *et al.* (2009) [84] Neutral temperature condition	11 men: Age = 20.8 (2.1); BMI = 24.1 (2.3); VO_2_ max = 53.8 (8.9) ml/kg/min	Control: 40 min rest. Exercise: 40 min treadmill running at 70% VO_2_ peak.	Buffet breakfast offered ∼30 min post trial.	EEEx = 2375 (280) kJ	EI was significantly higher following exercise vs. control. No significant difference in REI between exercise and control. No significant difference for PRO, fat or CHO intake as percentage of total EI.
Staten *et al.* (1991) [93]	10 men, 10 women: Age = NR; BMI = 22.1 (NR); VO_2_ max men = 47.3 (6.2) ml/kg/min; VO_2_ max women = 36.9 (6.0) ml/kg/min	5 day protocol with 2 conditions: Sedentary: Usual daily activities. Active: 60 min of daily treadmill exercise at 70% VO_2_ max	All food obtained from a metabolic kitchen.	EEEx for men = 669 (126) kcal/day. EEEx for women = 441 (92) kcal/day.	Significant increase in EI for men, but not women, in the exercise vs. the sedentary condition. However, REI estimates indicated that both men and women were in negative caloric balance during the exercise period.
Tremblay *et al.* (1994) [95]	9 men: Age = 28.3 (6.1); BMI = 23.9 (NR); VO_2_ max = 54.7 (6.6) ml/kg/min	Control: 60 min rest. Exercise: 60 min treadmill walk at 55–60% VO_2_ max.	EI over 48 hrs post trial was assessed when participants were offered low fat, high fat or mixed diet obtained from a metabolic kitchen.	EEEx = 2.8 (0.4) MJ	EI following exercise for both the low-fat and mixed-diet conditions were not different from control. However, when subjects ate the high-fat diet ad lib following exercise EI increased about 5.5 MJ/48 hr.
Tsofliou *et al.* (2003) [72]	10 women: Age = 50.0 (8.5); BMI = 37.2 (6.5); Activity level/fitness = NR	Control: 30 min sitting. Exercise: 29 min brisk walk indoors, under supervision at ∼13 on the RPE scale.	Buffet dinner presented 1-hr post trial	EEEx = NR. Exercise HR was 123 (18) bests/min at an RPE of 14 (2).	No significant difference in EI or macronutrient intake between trials.
Ueda *et al.* (2009) [85]	10 men: Age = 23.4 (4.3); BMI = 22.5 (1.0); VO_2_ max = 45.9 (8.5) ml/kg/min	Control: 30 min sitting. Exercise: 30 min recumbent cycle, moderate intensity (50% VO_2_ max). Exercise: 30 min recumbent cycle, high intensity (75% VO_2_ max).	Test meal offered 30 min post trial.	EEEx = NR	EI was significantly lower following both high and low intensity exercise vs. control. EI was not different between the moderate and high intensity trials.
Ueda *et al.* (2009) [86]	14 men: 7 normal weight, 7 obese. Normal weight: Age = 22.4 (4.2); BMI = 22.4 (2.4); VO_2_ max = 46.6 (3.9) ml/kg/min. Obese: Age = 22.9 (3.4); BMI = 30.0 (3.1); VO_2_ max = 34.0 (6.3) ml/kg/min	Control: 60 min sitting. Exercise: 60 min cycle ergometer at 50% VO_2_ max.	Test meal offered 2 hrs post trial.	EEEx = NR	EI and REI was significantly lower following exercise vs control session. There were significant differences in EI and REI between normal weight and obese males, suggesting a greater energy deficit due to exercise in obese compared with normal weight men.
Unick *et al.* (2010) [53]	19 sedentary women: Age = 28.5 (8.3 ); BMI = 32.5 (4.3)	Control: ∼40 min rest. Exercise: Treadmill walk at 70–75% of age-predicted max HR with a duration to elicit EEEx of 3 kcal/kg body weight.	Buffet of a variety of snack foods offered 60 min post trial.	EE rest = 44.3 (8.9) kcal. EEEx = 353.6 (71.9) kcal.	EI was not significantly different between control and exercise trials. REI was significantly lower following the exercise vs control.
Vatansever-Ozen *et al.* (2011) [80]	10 men: Age = 20.1 (0.2); BMI = 23.0 (0.4); VO_2_ max = 62.7 (5.1) ml/kg/min	Control: 4- hrs rest. Exercise: 120 min total – 105 min at 50% max VO_2_ and 15 min at 70% max VO_2_.	Buffet meal offered 1-hr post trial.	EEEx = 230 (20) kcal.	EI was not significantly different between trials.
Verger *et al.* (1992) [67]	5 active women: Age = Range 20–25; BMI = 19.5 (1.7). 8 active men: Age = Range 20–25; BMI = 23.4 (1.5)	Control: 3 hrs rest. Exercise: 2 hrs of non-stop athletic activities of similar intensity planned to be sub-maximal and aerobic.	Test meal offered immediately post trial or 30, 60 or 120 min post trial.	Estimated EEEx = 500 kcal.	EI for the meal consumed 60 min post exercise was significantly greater than control. EI increased as time post exercise increased. The increases from immediately post to 60 min post exercise and from immediately post to 120 min post exercise were statistically significant. Males and females responded similarly.
Verger *et al.* (1994) [83]	58 active men: Age = Range 18–22; BMI = 21.3 (1.6)	Participants were randomly assigned to: Control (n = 30): 2 hrs rest. Exercise (n = 28): 2 hrs of non-stop, submaximal athletic activities.	Buffet meal offered 30 min post trial.	Estimated EE control = 100 kcal/hr. Estimated EEEx = 400 kcal/hr.	EI post exercise was significantly higher than control.
Visona *et al.* (2002) [62]	36 sedentary women: Age = 26.0 (7.0); BMI = 27.0 (3.0). 3 groups of 12 representing dieting status and dietary restraint: 1. Dieter/High Restraint (D-HR) 2. Non-Dieter/High Restraint (ND-HR).3. Non-Dieter/Low Restraint (ND-LR).	Control: 60 min sitting. Exercise: 60 min treadmill walk at 60–70% max HR.	Cafeteria lunch offered 30 min post trial. 12 hr EI included the lunch plus all food consumed until bedtime on the experimental day assessed by food record.	EEEx = NR	There was a significant main effect of dieting/restraint status on lunch EI. The ND-LR group ate significantly more across the 2 trials than the D-HR group. There was a significant interaction of dieting/restraint status and condition (exercise vs. control) on 12-hr EI. The mean difference in 12-hr EI between the exercise and control day was significantly higher for the D-HR compared with the ND-HR group.
Whybrow *et al.* (2008) [88]	6 sedentary to moderately active women: Age = 24.7 (5.9); BMI = 22.9 (1.6). 6 sedentary to moderately active men: Age = 29.7 (5.9); BMI = 24.2 (2.2)	Participants were resident in, but not confined to a human nutrition research facility for 16 days (2 days run in, 14 days active intervention). Control: Usual activity. Moderate exercise = 2×40 min sessions on a cycle ergometer to expend 28.6 kJ/kg body weight. High exercise = 3×40 min sessions on a cycle ergometer to expend 57.1 kJ/kg body weight.	All meals selected from a menu of foods prepared by the research facility.	Women: EEEx moderate trial = 2.0 MJ/day. EEEx high trial = 3.8 MJ/day. Men: EEEx moderate trial = 2.8 MJ/day. EEEx high trial = 4.9 MJ/day.	Women: No significant between trial differences in average daily EI or macronutrient composition. Men: Average daily EI and energy from carbohydrate, fat and protein increased significantly with increased EEEx.

*Note*, Values are means (standard deviation) unless otherwise noted. Abbreviations: CHO = carbohydrate; EEEx = exercise energy expenditure; EI = energy intake; NR = not reported; PRO = protein; REI = relative energy intake; kcal = kilocalories; MJ = megajoules; KJ = kilojoules; hrs = hours; min = minutes; wk = week.

### Acute Studies: Study Characteristics

#### Sample size

The median (range) sample size across the 40 acute studies was 12 (7–65).

#### Exercise intensity/duration

The median (range) exercise intensity was 70% (60–75%) of HRMax, and 70% (30–75%) of maximal oxygen uptake. Five studies did not provide information on exercise intensity. The median (range) exercise duration was 50 min (3–90 min). Four acute studies dosed exercise by level of energy expenditure [51]–[54].

#### Energy intake assessment

Single ad-libitum test meals with energy intake assessed by the weigh and measure technique were utilized in the majority (25/40, 63%) of acute studies. Multiple ad-libitum meals [52], [55]–[58], a combination of ad-libitum meal plus energy intake over the rest of the day by recall [54], [59]–[62], diet recalls alone [63], and the consumption of specific food items, e.g. sandwiches [64], pasta salad [65] or cookies and chips [66] were also utilized.

#### Interval between end of exercise and energy intake assessment

Eleven acute trials assessed energy intake over the entire exercise day [52], [54]–[63]. In the remaining 29 acute studies, energy intake was assessed on one occasion with a median (range) of 30 minutes (immediate-330 min) post-exercise.

### Acute Studies: Participant Characteristics

#### Age

The median (range) age across the 36 studies that reported age was 23 (20.1–50) years.

#### Gender

Six studies included both men and women [64]–[68], 15 included women only [52]–[54], [62], [63], [69]–[76] and 19 studies included only men [51], [55]–[61], [77]–[86].

#### BMI

The median (range) BMI across 39 studies that reported BMI was 22.9 (19.8–37.2) kg/m^2^. Five of 40 studies (∼13%) included overweight or obese participants [53], [66], [72], [73], [85], [86].

#### Minority status

No studies described the racial or ethnic composition of the study sample or reported post-exercise energy intake by race or ethnicity.

#### Participant activity level

Participants recruited for the majority of acute studies (24/40–60%) were physically active and/or aerobically fit [51], [52], [54]–[60], [65], [67], [70], [71], [74], [76], [77], [79]–[84]. Eleven studies (∼28%) recruited sedentary or moderately active participants [53], [62], [63], [66], [68], [69], [73], [75], [85], [86] while 5 studies (∼13% ) did not describe baseline participant physical activity [61], [64], [72], [78].

### Acute Studies: Results

#### Energy intake

Nine of 40 acute studies (∼23%) [52], [62], [64], [65], [67], [68], [70], [83], [84] reported a significant increase in absolute energy intake (∼80 to 470 kcal/day) following exercise compared with non-exercise control while 27 studies (∼68%) found no difference in absolute energy intake between exercise and control conditions [51], [53]–[61], [63], [64], [66], [69], [72]–[77], [79]–[82]. Four studies (10%) reported a significant decrease in absolute energy intake (∼125 to 240 kcal/day) following exercise compared with non-exercise control [75], [78], [85], [86]. Fifteen studies (∼38%) reported a significant decrease in relative energy intake (energy intake - exercise energy expenditure) following exercise compared with control [51]–[53], [55], [57]–[59], [65], [68], [70], [71], [76], [79], [84]–[86]; 5 of those studies also reported significant increases in absolute energy intake [52], [65], [68], [70], [84] suggesting only partial compensation in energy intake following acute exercise.

#### Macronutrient intake

Sixteen of the 40 acute studies (40%) reported data on macronutrient intake [54]–[58], [60], [68], [72], [76]–[79], [82], [84]. Fifteen studies showed no effect, while one study indicated significantly higher fat and protein intake following exercise compared with non-exercise control [76].

### Effect of Study Parameters on Energy Intake

#### Exercise mode

Three studies provided information relative to the effect of exercise mode on post-exercise energy intake. Balaguera-Cortes *et al.* [77] reported no effect of either aerobic (treadmill) or resistance exercise while King *et al.* [56] showed no effect of swimming on absolute post-exercise energy intake. These results are in contrast to those of Laan *et al.* [65] who showed an increase in absolute post-exercise energy intake following both aerobic (cycling) and resistance exercise; however, relative energy intake was lower following aerobic exercise compared to resistance exercise or control.

#### Exercise intensity

Six acute studies reported the effect of exercise intensity on post-exercise energy intake. Four studies found no effect of exercise intensity on absolute energy intake following exercise [51], [60], [64], [85]; however, Imbeault *et al.* [51] reported a lower relative energy intake following high intensity exercise (75% VO_2_ max) compared with low intensity exercise (35% VO_2_ max) or non-exercise control. One study [52] showed a significant increase in absolute energy intake for high (70% VO_2_ peak) but not low intensity exercise (40% VO_2_ peak); however relative energy intake was lower in both the high and low intensity exercise groups compared with non-exercise controls. One study reported a significant decrease in absolute energy intake following strenuous (40 min/90 W cycle ergometer) but not moderate exercise (40 min/30 W cycle ergometer) in non-obese but not in obese women [75].

#### Exercise duration

Two studies evaluated the role of exercise duration on post-exercise energy intake with divergent results. King *et al.* [60] reported no effect of exercise duration on post-exercise energy intake; however, Erdman *et al.* [64] reported that absolute energy intake was not significantly greater than control following cycle ergometer exercise bouts of 30 and 60 min, but was significantly greater than control following 120 minutes of exercise.

#### Exercise time of day

Two studies evaluated the effect of the time of day of aerobic exercise on post-exercise energy intake [55], [63]. Both studies found no significant difference in absolute post-exercise energy intake between exercise performed in the morning (7 and 8:15 AM) compared to the same exercise performed in the evening (5 and 7:15 PM). However, O'Donoghue *et al.* [55] showed that relative energy intake at breakfast was lower after morning exercise compared with afternoon exercise or control while relative energy intake at dinner was lower post afternoon exercise compared with control.

#### Composition of test meals

Four studies evaluated the effect of the macronutrient composition of the test meal on post-exercise energy intake. Three studies found no significant differences in absolute post-exercise energy intake compared to rest between low or high fat test meals [70], [71], [74]. King *et al.* [59] found no difference in absolute post-exercise energy intake when either high fat/low carbohydrate or low fat/high carbohydrate test meals were presented; however, relative energy intake was significantly lower in the low fat/high carbohydrate, but not the high fat/low carbohydrate condition compared with control.

#### Time between the end of exercise and the presentation of the test meal

In the one acute study that investigated the effect of time between exercise and presentation of the test meal on energy intake, Verger *et al.* [67] showed that absolute energy intake increased as the time post-exercise that the test meals were presented increased (immediate to 120 min).

### Effect of Participant Characteristics on Energy Intake

#### Age

No studies evaluated the effect of age on post-exercise energy intake. Studies were generally conducted in young adults with a median age of 23 years.

#### Gender

Although 6 studies included both men and women [64]–[68] the data were presented separately in only one study. Verger *et al.* [67] showed significant increases in absolute EI following exercise (2 hours of non-stop submaximal aerobic athletic activities) in both men and women.

#### Weight status

Three studies provided data on the effect of weight status on post-exercise energy intake [73], [75], [86]. George *et al.* [73] found non-significant differences in absolute post-exercise energy intake between normal and overweight women. Kissileff *et al.* [75] reported significant decreases in post-exercise energy intake in non-obese, but not obese women, while Ueda *et al.* [86] found larger energy deficits (i.e. decreased energy intake) induced by exercise in obese compared with normal weight men.

#### Weight/Diet/Dietary restraint

Three studies evaluated the effect of combinations of weight, dieting status or level of eating restraint on post-exercise energy intake. Harris *et al.* [61] found no differences in post-exercise energy intake in a sample of men across 5 groups: 1) normal weight/low dietary restraint/non-dieting; 2) normal weight/high dietary restraint/non-dieting; 3) overweight/low dietary restraint/non-dieting; 4) overweight/high dietary restraint/non-dieting; and 5) overweight/high dietary restraint/dieting. In a sample of normal weight young women, Lluch *et al.* [70] found increased absolute post-exercise energy intake in women classified as unrestrained eaters and decreased energy intake in restrained eaters. Relative energy intake compared with rest was greater in restrained compared with unrestrained eaters. Visonia *et al.* [62] demonstrated a significant interaction between dieting/eating restraint status and study condition (exercise vs. control) on 12-hour energy intake in sample of women. The mean difference in 12-hour energy intake between the exercise and control day was significantly higher for the dieting-high restraint group compared with the non-dieting high restraint group.

#### Activity level

Two studies evaluated the effect of activity level on post-exercise energy intake. Jokisch *et al.* [78] showed a significant decrease in post-exercise energy intake compared with control in inactive but not in active men. Larson-Meyer *et al.* [76] found non-significant differences between post-exercise energy intake and control in a normal weight sample of both habitual walkers (≥3 days/wk for ≥60 min/day) and habitual runners (≥32 km/wk); however, relative post-exercise energy intake was significantly lower in runners compared with controls, but not in walkers vs. controls.

### Short-Term Studies

The 10 short-term studies comprised ∼9% of the total studies identified for this review (Table 2). These studies employed cross-over designs that compared energy intake assessed over a time frame of 2–14 days during which participants engaged in exercise with energy intake during an equivalent period of no imposed exercise.

### Short-Term Studies: Study Characteristics

#### Sample size

The median (range) sample size for the 10 short-term studies was 9.5 (6–20)

#### Trial length

The median (range) duration of short-term studies was 8 (2–14) days.

#### Exercise intensity/duration

One study prescribed exercise intensity at 70% of heart rate max (HRMax). Five studies prescribed intensity relative to VO_2_ max (median [range] 60% [44–75%]). Three studies did not report exercise intensity relative to VO_2_ or HRMax [19], [87], [88]. Eight of 10 short-term studies (80%) dosed exercise by energy expenditure; 3 relative to body weight [87]–[89], 3 relative to resting or baseline daily energy expenditure [90]–[92], and 2 to an absolute exercise energy expenditure goal [93], [94]. Two studies prescribed exercised by time that ranged from 60 [93] to 100 min/day [94]. Prescriptions ranged from 21.4 [89] to 57.1 kJ/kg body weight [88], 1.4 to 1.8 times RMR [92], 12.5% [90] to 29% above baseline total energy expenditure [91] and net exercise energy expenditure from 2.8 [95] to 2.98 MJ/day [96].

#### Energy intake assessment

Seven studies used weigh and measured meals provided by a metabolic kitchen [88], [90]–[93], [95], [96] 3 used weigh and measure food records [87], [89], [94].

### Short-Term Studies: Participant Characteristics

#### Age

The median (range) for age across all short-term studies was 28.3 (23–35) years.

#### Gender

Two studies (20%) included both men and women [88], [93], 3 (30%) included women only [87], [90], [91] and 5 studies (50%) included only men [89], [92], [94]–[96].

#### BMI

The median (range) BMI was 23.5 (21.4–28.2) kg/m^2^. Three of 10 studies (30%) had a mean sample BMI in the overweight category (i.e. ≥25 kg/m^2^) [90], [91], [96].

#### Minority status

No studies describe the racial or ethnic composition of the study sample or reported an association between exercise level and energy intake by race or ethnicity.

#### Participant activity level

Participants recruited for the majority of short-term studies (7/10 - 70%) were sedentary or moderately active [87]–[90], [92], [93], [96]. Two studies (20%) recruited active participants [94], [95] while one study (10%) did not describe baseline participant physical activity [91].

### Short-Term Studies: Results

#### Energy intake

Five of 10 short-term studies (50%) reported increased absolute energy intake (∼200–335 kcal/day) over periods of 2 to14 days when exercise was imposed compared with a non-exercise control period [87], [88], [93], [95], [96]. Three studies that reported increased absolute energy intake showed relative energy intake at a level to maintain a negative energy balance during the exercise period [87], [88], [93]; however, Tremblay et al [95] showed that participants achieved a positive energy balance when presented with a high fat diet.

#### Macronutrient intake

Seven of the 10 short-term studies (70%) reported macronutrient intake [87]–[90], [92], [94], [96]. Four of 7 studies (57%) showed no effect of exercise on macronutrient intake [89], [90], [92], [94]. Farah *et al.* [96] reported increased intake of carbohydrate and protein while Stubbs *et al.* [87] observed increased intake of carbohydrate and fat with exercise compared to control. Whybrow *et al.* [88] noted increased intake of carbohydrate, fat and protein with exercise vs. control in men but not in women.

### Effect of Study Parameters on Energy Intake

#### Exercise mode

The one study that compared the effect of aerobic and resistance exercise reported no significant difference between exercise and control for short-term energy intake during either aerobic or resistance training [91].

#### Level of exercise energy expenditure

Four studies evaluated the effect of increased levels of exercise energy expenditure on short-term energy intake [87]–[90]. Levels of energy compared were 12.5% vs. 25% above baseline energy expenditure [90], 21.4 vs. 42.8 kJ/kg/day [87], [89] and 28.6 vs.57.1 kJ/kg/day [88]. Three studies observed no effect [87], [89], [90]. The study by Whybrow *et al.* [88] reported increased short-term energy intake associated with higher levels of exercise energy expenditure (57.1 vs. 28.6 kJ/kg/day) in men but not in women.

#### Exercise intensity

No studies evaluated the effect of exercise intensity on short-term energy intake.

#### Composition of test meals

The one study that compared the effect of the composition of test meals (mixed, high fat, low fat) on short-term energy intake noted increased energy intake when high fat, but not low or mixed fat meals were presented [95].

### Effect of Participant Characteristics on Energy Intake

#### Age

No short-term studies evaluated the effect of age on post-exercise energy intake. Studies were generally conducted in young adults with a median age of 28.3 years.

#### Gender

Results from the two studies that evaluated gender differences in short-term energy intake with exercise reported that absolute energy intake increased in men but not in women [88], [93]. Results from 2 separate studies that used identical exercise and energy intake protocols in samples of men [89] and women [87] found increased energy intake with exercise in women, but not in men.

#### Other participant characteristics

No studies evaluated the effect of weight, physical activity or level of dietary restraint on short-term changes in energy intake induced by exercise.

### Non-Randomized Trials

The 12 non-randomized trials comprised ∼12% of the total studies identified for this review (Table 3). Most trials (11/12) evaluated changes in energy intake in a single group (no control) assigned to complete a longitudinal exercise training program [18], [21], [97]–[105] while one study observed differences in energy intake between women who participated in an 8 week exercise program at a commercial exercise facility with a group of non-exercise volunteer controls [106].

**Table 3:** Non-Randomized Trials.

Study	Participants	Trial Length	Exercise Prescription/Intervention	Diet Assessment	Results: Exercise	Results: Diet
Andersson *et al.* (1991) [101]	22 women: Age = 36 (1.4) years; BMI = 24.6 (0.3); VO_2_ max = 33.9 (NR) ml/kg/min. 9 men: Age = 37 (2.4) years; BMI = 25.7 (0.4); VO_2_ max = 42.0 (2.8) ml/kg/min	12 wks	Supervised 60 min sessions of gym activity, 3 times/wk. Interval training - lighter intensity exercise (jogging, coordination, strength) with 3–5 min periods at 80% max work capacity.	Diet history at baseline, mid and end study.	EEEx or other data on exercise adherence were NR	No significant change in EI for lean women (n = 15, mean body fat 24.8 kg). EI was significantly decreased in obese women (n = 7, mean body fat 31.3 kg). No significant change in EI for men
Bryant *et al.* (2012) [103]	Total sample (39 women & 19 men): Age = 35.6 (9.8) years; BMI = 31.8 (4.5); VO_2_ max = 29.1 (5.7) ml/kg/min)	12 wks	Supervised sessions of cycle ergometer exercise, 5 days/wk at 70% VO_2_ max for a duration designed to expend 500 kcal/session.	EI intake was measured during probe days every 4 weeks. EI was calculated by weighing food before and after consumption.	EEEx or other data on exercise adherence were NR	No significantly change in EI over the course of the intervention.
Caudwell *et al.* (2013) [104]	72 women: Age = 40.6 (9.5); BMI = 31.8 (4.3); VO_2_ max = 28.9 (NR) ml/kg/min. 35 men: Age = 41.3 (8.6) years; BMI = 30.5 (8.6); VO_2_ max = 34.7 (NR) ml/kg/min	12 wks	Supervised exercise 5 days/wk at 70% HR max with a duration designed to expend ∼10.5 MJ/wk. Mode: choice of treadmill, cross-trainer, rowing or cycle ergometers.	Test meals in the lab at baseline and 12 wks. After an individualized fixed-energy breakfast (ad libitum on the first visit), participants were provided with a fixed-energy lunch, an ad libitum dinner meal, and a snack box for the evening.	Total EEEx over 12 wks was 122.8 (20.9) MJ for men and 115.3 (15.1) MJ for women, NS. Average exercise session for men was 43 (8.7) min and 54 (10.2) min for women.	No significant change in EI for either women. No significant change in EI for either men
Di Blasio *et al.* (2012) [105]	41 women (34 completed intervention). Completer data: Age = 55.9 (3.6); BMI = 26.9 (4.2); VO_2_ max = 27.0 (5.0) ml/kg/min	13 wks	Group walks, 4 days/wk. Month 1: 40 min at RPE 11 on 15 point scale. Month 2: 50 min/RPE 11. Month 3: 50 min/RPE 13. 2 of 4 sessions/wk were supervised.	3–day food records (2 weekdays and 1 weekend day) collected at baseline and 13 wks.	Attendance at exercise sessions was 85 (10.8)%. No significant increase in total or PA EE (SenseWear Armband) between baseline and 13 wks.	No significant change in EI or % of energy from fat, CHO or PRO.
Keytel *et al.* (2001) [106]	19 women (non-randomized observation of 9 exerciser and 10 non-exercisers). Exercisers: Age = 58 (7) years; BMI = 25.1 (2.7); VO_2_ max = 1.87 (0.42) L/min. Non-exercisers: Age = 55 (5) years; BMI = 26.6 (4.3); VO_2_ max = 1.96 (0.42) L/min.	8 wks	Exercise: New participants in a commercial walk/run exercise program; met 3 days/wk. Started walking 3 km and progressed to walk/jog between 3–6 km at 70–75% of age predicted max HR. Non-exercise: Volunteers recruited by media and investigator. Told no to make major lifestyle changes.	1–24 hr recall at baseline and 2 at 8 wks.	No difference in total daily EE across 8 wks. in either the exercise or control group. Activity diary data indicated significantly greater levels of PA in exercise vs. control for sedentary, active and very active categories.	No significant change in EI or % of energy from fat, CHO or PRO.
King *et al.* (2008) [18]	35 total (25 women &10 men): Age = 39.6 (11) years; BMI = 31.8 (4.1); VO_2_ max = 28.6 (5.7) ml/kg/min	12 wks	Supervised exercise, 5 days/wk at 70% max HR, duration to expend ∼500 kcal/session. Mode: cycle ergometer, stepper, rowing ergometer or treadmill.	Test meals (lunch/dinner) on 1 day at baseline and 12 wks.	Attendance at exercise sessions was 82%. EEEx was 2333 kcal/wk.	No significant change in EI or % of energy from fat, CHO or PRO.
Koulouri *et al.* (2006) [97]	12 total (7 women & 5 men) active participants enrolled.10 completers: Age = 28.3 (6.0) years; BMI = 22.3 (3.3).	3 wks	1 wk habitual activity followed by 2 wks of advice to increase walking by 2000 steps/day.	Daily food diaries over the 3 wk study period.	During the exercise period steps/day (pedometer) increased by 2, 677; EE (diary) increased 30 kJ/day, and EE (activPAL) increased 100 kJ/day.	No significant change in EI between baseline and the intervention period.
Manthou *et al.* (2010) [21]	34 women: Age = 31.7 (8.1) years; BMI = 29.3 (4.3); VO_2_ max = 2.1 (0.38) L/min.	8 wks	150 min of supervised cycle ergometer exercise at 72–77% of age-predicted max HR in one of 2 patterns: 75 min 2 days/wk (n = 18) or 30 min 5 days/wk (n = 16)	7-day weighed food record at baseline and 8 wks.	Compliance with exercise was 100%. EEEx = 30.2 (12.6 MJ) over the intervention.	EI increased significantly from baseline to 8 wks. No significant changes in energy from fat, CHO or PRO. Differences in EI or macronutrient intake between exercise patterns was not presented.
Martins *et al.* (2010) [98]	22 total (Completer: 7 women & 8 men): Age = 36.9 (8.3) years; BMI = 31.3 (2.3); VO_2_ max = 32.9 (6.6) ml/kg/min	12 wks	Supervised treadmill walking or running 5 days/wk at 75% max HR to induce and energy deficit of 500 kcal/session.	3-day food record (at least 1 weekend day) at baseline and 12 wks.	Average attendance at exercise sessions = 89 (5.9)%	No significant change in EI or macronutrient intake.
Snyder *et al* (1997) [99]	13 women (15 started): Age = 43 (11); BMI = 32.5 (8.0); VO_2_ max = 24.0 (4.6) ml/kg/min	32 wks	Walking (in and outdoors) 10 min bouts, 3 times/day, 5 days/wk. at 50–67% HRR. 3 of 5 sessions/wk were supervised.	3-day food record at baseline and 32 wks; 24- hr. recalls at 11 and 22 wks.	Adherence to exercise = 82.6 (10)%. Total estimated EEEx = 81843 (21014) kJ.	No significant change in EI or macronutrient intake.
Suzuki *et al.* (1998) [102]	31 women: Age = 19.2 (0.2 SEM) years; BMI = 21.5 (1.2 SEM); VO_2_ max = 36.2 (1.1) ml/kg/min	12 wks	Supervised cycle ergometer exercise, 30 min/day, 5 days/wk at 40% VO_2_ max.	Daily weighed food records analyzed weekly.	EEEx = 117.3 (3.1 SEM) kcal/session.	No significant change in EI or macronutrient intake between baseline and the training period.
Westerterp *et al.* (1992) [100]	32 total (16 women &16 men) sedentary participants enrolled: Age range = 28–41 years; BMI range = 19.4–26.4. 5 women and 4 men did not complete the intervention.	44 wks	Supervised training (running), 4 sessions/wk. With increasing running time to 10–30 min, 20–60 min and 30–90 min/session after 8, 20 and 40 weeks respectively. Goal of running a half marathon.	7-day weighed food records at baseline, 8, 20 and 40 wks.	Median PA index (daily EE/sleep EE) increased over the intervention.	No significant changes in or nutrient composition of the diet were detected.

*Note*, Values are means (standard deviation) unless otherwise noted. Abbreviations: CHO = carbohydrate; PA = physical activity; EE = energy expenditure; EI = energy intake; NR = not reported; PRO = protein; REI = relative energy intake; kcal = kilocalories; KJ = kilojoules; MET = Metabolic equivalent of task; hrs = hours; min = minutes; wk = week.

### Non-Randomized Trials: Study Characteristics

#### Sample size/completion rate

The median (range) sample size across the 12 non-randomized trials was 31 (10–107). The median (range) rate of trial completion in the 5 trials that provided data on this parameter was 83% (68–87%) of participants who started the intervention [97]–[100], [105].

#### Trial length

The median (range) length of non-randomized trials was 12 (3–44) weeks.

#### Exercise mode

Six of 12 non-randomized trials involved laboratory based aerobic exercise conducted on cycle ergometers/rowers/steppers/treadmills [18], [21], [98], [102]–[104], 5 trials employed indoor or outdoor walking [97], [99], [100], [105], [106]while one trial required participants to complete a combination of gym activities (jogging/coordination/resistance) [101].

#### Exercise supervision

All exercise sessions were supervised in 8 trials [18], [21], [98], [100]–[104], partially supervised in 3 trials [99], [105], [106] and unsupervised in one trial [97].

#### Exercise prescription (frequency)

The median (range) exercise frequency was 5 (2–5) days/week.

#### Exercise prescription (intensity)

Three trials prescribed intensity as a percentage of maximal VO_2_ [101]–[103], 5 by percentage of HRMax [18], [21], [98], [104], [106], one by heart-rate-reserve [99] and one by ratings of perceived exertion [105]. The median (range) of intensity prescriptions were: 40% (40–80%) max VO_2_; 73% (70–75%) HRMax, 59–67% heart-rate-reserve; and perceived exertion 11–13 on a 15 point scale. Prescribed exercise intensity was not reported in 2 trials [97], [100].

#### Exercise prescription (duration)

Six trials prescribed exercise duration by time [21], [99]–[102], [105], 4 by level of exercise energy expenditure [18], [98], [103], [104], one by walking distance [106] and one by pedometer steps/day [97]. The median (range) duration for the 6 trials prescribing exercise by time was 40 (30–60) min/day. All 4 studies prescribing exercise by energy expenditure assigned 500 kcal/exercise session. Prescribed walking distance was 3–6 km/day, and pedometer steps were to increase steps by 2,000 per day above baseline over 2 weeks.

#### Compliance with the exercise protocol

Seven studies presented data relative to participant compliance with the exercise protocol [18], [21], [97]–[99], [104], [105]. Five trials reported the percentage of exercise sessions attended (range 82–100%) [18], [21], [98], [99], [105], 1 reported the level of exercise energy expenditure (prescribed 10.5 MJ/wk; achieved 9.9 MJ/wk) [104] and one trial reported pedometer steps/day (prescribed 2,000; achieved 2,677 steps/day) [97].

#### Energy and macronutrient assessment

Five trials used non-weighed food records [97], [98], [100], [101], [105], 2 used weighed food records [21], [102], 3 used test meals [18], [103], [104], 1 used 12 hour recall [106] and one used a combination of food records and 24-hour recalls [99]. Six studies assessed energy intake at baseline and end [18], [21], [98], [101], [104], [105], 3 studies completed energy intake assessments at 4 time points [99], [100], [103], one study at 3 time points [106] while 2 studies collected daily estimates of energy intake over the course of the intervention [97], [102].

### Non-Randomized Trials: Participant Characteristics

#### Age

The median (range) age across all non-randomized trials was 36.9 (19.2–56) years.

#### Gender

Seven studies (58%) included both men and women [18], [97], [98], [100], [101], [103], [104] while 5 studies (42%) included women only [21], [99], [102], [105], [106].

#### BMI

The median (range) BMI was 29.3 (21.5–32.5) kg/m^2^. Five of 12 studies (42%) had a mean sample BMI in the overweight category (i.e. ≥25 kg/m^2^) [21], [100], [101], [105], [106], while the mean sample BMI was classified as obese (i.e. ≥30 kg/m^2^) in 5 trials [18], [98], [99], [103], [104] and normal weight (i.e. BMI<25 kg/m^2^) in 2 trials [97], [102].

#### Minority status

No non-randomized trials described the racial or ethnic composition of the study sample or reported an association between exercise level and energy intake by race or ethnicity.

#### Participant activity level

Eleven of 12 non-randomized trials described inclusion criteria for level of baseline physical activity or aerobic fitness. With the exception of the trial of Koulouri *et al.* [97] who recruited regularly active participants, non-randomized trials were conducted in participants categorized as sedentary [18], [21], [98], [100], [102], [103], [105], [106]or with low aerobic fitness [99], [104].

### Non-Randomized Trials: Results

#### Energy intake

Eleven of 12 (92%) of non-randomized trials reported no change in energy intake in response to exercise training [18], [97]–[106]. One non-randomized trial reported a significant increase in energy intake (∼84 kcal/day) as a result of participating in an exercise training program [21].

#### Macronutrient intake

None of the 8 non-randomized trials that evaluated the effect of exercise training on macronutrient intake reported significant change in the intake of carbohydrate, fat or protein [18], [21], [98]–[100], [102], [105], [106].

### Effect of Study Parameters on Energy Intake

#### Exercise mode

No studies evaluated the effect of exercise mode on changes in energy intake in response to exercise training.

#### Level of exercise energy expenditure/duration

No studies evaluated the effect of exercise energy expenditure/duration on energy intake in response to exercise training.

#### Exercise intensity

No studies evaluated the effect of exercise intensity on energy intake in response to exercise training.

#### Composition of test meals

Three non-randomized trials evaluated energy intake using test meals [18], [103], [104]; however, the effect of variations in the macronutrient composition of the test meals was not evaluated.

### Effect of Participant Characteristics on Energy Intake

#### Age

No studies evaluated the effect of age on changes in energy intake in response to exercise training.

#### Gender

Three non-randomized trials provided data on gender differences [100], [101], [104]. Two trials reported no differences for change in energy intake between men and women in response to exercise training [100], [104], while one trial found no effect in men or lean women, and a significant decrease in energy intake with exercise training in obese women [101].

#### Other participant characteristics

No non-randomized trials were identified that specifically evaluated the effect of weight status, level of physical activity or level of dietary restraint on the energy intake response to exercise training.

### Randomized Trials

The 24 randomized trials constituted ∼24% of the total number of studies identified for this review (Table 4). The majority of trials (16/24; ∼67%) evaluated the effect of exercise training on energy and macronutrient intake between participants randomized to exercise compared with non-exercise controls [107]–[122]. The effect of specific exercise training parameters on energy intake including mode (resistance/resistance plus aerobic/swim) [118]–[120], [123], [124], intensity [124]–[127], volume [116], [121], [122] and timing (intermittent vs. continuous) [128] were also reported. The majority of randomized trials employed an efficacy design; however, reports from Jakicic *et al.* [122] and Foster-Schubert *et al.* [110] employed an intent-to-treat design, and Rosenkilde *et al.* [121] reported both efficacy and intent-to-treat results.

**Table 4:** Randomized Trials.

Study	Participants	Length	Intervention/Groups	Diet Assessment	Results: Exercise	Results: Diet
Bales *et al.* (2012) [123]	117 sedentary men and women in 3 groups: Aerobic training (AT) 19 men/20 women: Age = 53 (NR); BMI = 30.1 (NR); Resistance training (RT) 18 men/20 women: Age = 48.5 (NR); BMI = 30.4 (NR). Aerobic+Resistance training (AT/RT) 18 men/22 women: Age = 47 (NR); BMI = 30.2 (NR)	8 mos	AT: Calorically equivalent to approximately 12 miles/wk at 65%–80% VO_2_ peak) treadmill, elliptical and cycle ergometer. RT: 3 days/wk., 3 sets, 8–12 reps. RT program began with one set of each exercise during the first 2 wks., followed by two sets during weeks 3 and 4, and reaching the goal of 3 sets at week 5. AT/RT: Linear combination of both exercise prescriptions.	2 EI assessment methods were used at baseline and end study: 1) Combination of 3-day food records (2 wk days/1 wk end day) and unannounced 24 hr. recall. (QDI). 2) Block Food Frequency Questionnaire (FFQ).	Adherence to exercise treatment: AT = 90.1%; RT = 82.9%; AT/RT = 83.3%/82.0%	FFQ results: Significant decreases in absolute EI, and the intake of CHO, fat and PRO for both the AT and AT/RT groups. QDI results: Changes were non-significant; except a significant decreased in absolute fat intake for RT. When EI was expressed relative to body mass, results were similar, i.e., significant decreases EI for AT and AT/RT (FFQ results), with no change in RT.
Brandon & Elliott-Lloyd (2006) [107]	52 women: Exercise = 15 African American: Age = 34 (7.2); BMI = 34.4 (8.2); VO_2_ max = 31.4 (5.2) ml/kg/min; 13 White: Age = 40.5 (7.1); BMI = 29.5 (5.7); VO_2_ max = 32.3 (6.0) ml/kg/min. Control = 12 African American: Age = 36 (8.4); BMI = 33.0 (7.1); VO_2_ max = 30.8 (4.5) ml/kg/min. 12 White: Age = 42 (9.7); BMI = 32.8 ( 7.3); VO_2_ max = 32.3 (6.0) ml/kg/min	2 wk run-in period, 16 wks at walking goal. Studied only women who were able to walk 3 miles/session after the 2 wk run-in period.	Control: No exercise. Exercise: Supervised brisk walking in groups, 3 days/wk., 3 miles/session, outdoors (indoor track or treadmill in bad weather). Self-paced intensity. Goal was to walk 3.5 mph pace.	2-day food record (Sunday and Monday) at study wks. 1, 9 and 16.	Exercise Adherence: African American = 86%; White = 90%. Pace: African American = 3.54 mph; White = 3.61 mph. Retention rate was higher in Whites (74%) vs. African American (45%) based on performance in the run-in period.	African American: EI significantly increased from baseline to wk 16. CHO intake (g/day) increased, no change in fat or PRO intake. Whites: Reductions in EI below baseline at both wks 9 (12.7%) and wk. 16 (5.1%) were observed, statistically significant at wk. 8 only. CHO intake decreased significantly (wk. 9), no change in fat or PRO intake.
Broeder *et al.* (1992) [108]	64 men randomized. Control, n = 20; Endurance, n = 22; Resistance, n = 22. 47 men completed the study. Control n = 19; Age = 18–35; BMI = 25.3 (1.0); VO_2_ max = 49.1 (2.2) ml/kg/min. Endurance, n = 15; Age = 18–35; BMI = 25.1 (1.1); VO_2_ max = 49.6 (2.2) ml/kg/min. Resistance, n = 13; Age = 18–35; BMI = 25.5 (1.1); VO_2_ max = 48.1 (1.5) ml/kg/min	12 wks	Endurance training: Supervised walk/jog program, 4 days/wk progressed to 40 min at a minimum intensity of 70% VO_2_ max by wk 4. After wk 8, each person was exercising for 50 min at between 70% and 85% VO_2_ max. Resistance training: Supervised free weights/machines, 4 days/wk. (2 days upper and 2 days lower body), 3-sets, 11 exercises.	3-day food records at baseline, and wks 6, 7 and 12. Recording days randomly assigned but always included 2 week days and 1 weekend day.	Exercise groups each subject completed ≥90% of scheduled workout sessions.	No significant change in EI for any study group. No significant between group differences between the mid and post-treatment periods for the percentage of kcal derived from CHO, fat or PRO.
Bryner *et al.* (1997) [125]	25 women randomized, 15 women completed study. Low intensity = 7 women: Age = 24.9 (4.6); BMI = 23.6 (NR); VO_2_ max = 36.2 (4.7) ml/kg/min. High intensity = 8 women: Age = 24.2 (6.0); BMI = 23.2 (NR); VO_2_ max = 29.2 (5.7) ml/kg/min	16 wk trial, 12 wk active intervention.	Supervised walk/jog, 40–45 min/day, 4 days/wk at either low or high intensity. Low intensity: 60–70% max HR. High intensity: 80–90% max HR	7-day food records, monthly.	Low intensity group: Exercise day/wk = 4.2 (0.47). Min/day = 46 (10.2); HR = 131.6 (7.6). High intensity group: Exercise day/wk = 3.9 (0.3); Min/day = 43 (9.3); HR = 162.8 (9.5)	No significant change in EI or CHO, fat or PRO intake for either group.
Cox *et al.* (2010) [124]	116 women. Walk group (60 women): Age = 55.2 (4.8); BMI = 26.4 (3.5); VO_2_ max = 27.5 (4.1) ml/kg/min. Swim group (56 women): Age = 55.8 (4.5); BMI = 26.3 (3.0); VO_2_ max = 27.5 (4.2) ml/kg/min. 42 walk and 44 swim group completed 12 mos. assessments for a retention rate of 74%.	12 mos	6 mos. supervised and 6 mos. unsupervised exercise. Walk: 30 min/session, 3 days/wk. at 60–70% HRR, on a track or around a park. Swimming: 30 min/session, 3 days/wk. at 60–70% HRR. Unsupervised exercise at a venue of choice. Half of women received a behavioral intervention package to encourage adoption and adherence to exercise, the other half received standard exercise information of “usual care”	FFQ at baseline, 6 and 12 mos which assessed nutrient intake for the previous 3 mos.	Exercise min/wk. at 6 mos. Walk = 122 min/wk. Swim = 126 min/wk. Exercise min/wk at 12 mos. Walk = 116 min/wk.Swim = 117 min/wk.	EI was unchanged, with no significant between-group differences after 6 or 12 mos.
Cox *et al.* (2003) [129] Normal Diet Arm	Light exercise (17 men): Age = NR; BMI = 29.7 (3.4); VO_2_ max = 2.4(range 2.2–2.6) L/min. Vigorous exercise (13 men): Age = NR; BMI = 30.5 (3.9); VO_2_ max = 2.6 (range 2.4–2.8) L/min. Mean age total sample = 42.4 (5.0).	16 wks	Supervised exercise 30 min/session, 3 days/wk. Light exercise: Slow flexibility exercises 1 day/wk. and stationary cycling (zero resistance) 2 days/wk. Vigorous exercise: Stationary cycling at 60%–70% HRR.	3-day food records completed every 2 wks.	Exercise intensity: Light exercise = 18% HRR. Vigorous intensity = 76% HRR. EEEx ranged from 1,507 to 1,758 kJ/session.	No significant change in EI or % of energy from CHO, fat or PRO in either the light or vigorous exercise groups.
Church *et al.* (2009) [16]	411 women. Control (94 women): Age = 57.2 (5.9); BMI = 32.2 (3.9); VO_2_ max = 15.7 (2.0) ml/kg/min. Exercise 4 kcal/kg/wk (KKW):139 women: Age = 57.9 (6.5); BMI = 31.4 (3.7); VO_2_ max = 15.5 (2.9) ml/kg/min. Exercise 8 KKW (85 women): Age = 56.7 (6.4); BMI = 32.2 (4.1); VO_2_ max = 15.1 (2.2) ml/kg/min. Exercise 12 KKW (93 women): Age = 56.4 (6.3); BMI = 31.1 (3.6); VO_2_ max = 16.1 (2.8) ml/kg/min	6 mos	Control: no exercise. Exercise groups: 3–4 sessions/wk. at 50% VO_2_ max designed to achieve EEEx of 4, 8 and 12 KKW. Exercise alternated between semi-recumbent cycle ergometers and treadmills.	EI at baseline and end study was assessed using the Food Intake and Analysis System semi-quantitative food frequency questionnaire.	Exercise adherence: 4 KKW = 99.5%; 8 KKW = 99.3%; 12 KKS = 99.2%. Exercise min/wk.: 4 KKW = 72.2 (12.4); 8 KKW = 136.3 (19.4); 12 KKW = 193 (31). Total EEEx completed (kcal): 4 KKW = 7932; 8 KKW = 15197; 12 KKW = 20560	No significant between group differences in mean EI at either baseline or end study. For all groups the mean EI was significantly lower at end study compared to baseline. There were no between groups difference in baseline or follow-up in dietary PRO, fat and CHO intake.
Donnelly *et al.* (2003) [9]	131 men/women randomized; 74 men/women completed the study (56.5%). Control = 15 men: BMI = 29.0 (3.0); VO_2_ max = 39.5 (5.7) ml/kg/min.18 women: BMI = 29.3 (2.3); VO_2_ max = 32.4 (3.1) ml/kg/min. Exercise = 16 men: BMI = 29.7 (2.9); VO_2_ max = 39.2 (5.2) ml/kg/min.25 women: BMI = 28.7 (3.2); VO_2_ max = 32.8 (4.2) ml/kg/min. Sample age NR: recruited age range 17–35.	16 mos	Control: no exercise. Exercise: supervised treadmill exercise, 5 days/wk. progressed from 20 min/session at baseline to 45 min/session at 6 mos. and intensity progressed from 60% HRR at baseline to 75% HRR at 6 mos.	Weight and measure over 2 wk periods at baseline, 3, 6, 9, 12 and 16 mos. of ad libitum eating at a university cafeteria. Food consumption outside the cafeteria (i.e. snacks) was assessed by multiple-pass 24-hr recalls.	Exercise adherence: Men = 90.3%; Women = 89.6%. EEEx: Men = 667 (116) kcal/session. Women = 439 (88) kcal/session	Men: No significant between group differences at baseline or at 16 mos. for EI, fat (g), or CHO (g) intake. PRO intake (g) increased significantly in men in the exercise group vs control. No significant between group differences were observed for fat, CHO or PRO intake as a percentage of total EI. Women: No significant between group differences at baseline or 16 mos for EI or either absolute (g) or relative (% EI) intake of fat, CHO, or PRO.
Donnelly *et al.* (2000) [128]	22 women. Continuous exercise (CON) = 11 women: Age = 54 (9); BMI = 30.1 (2.5); VO_2_ max = 23.6 (2.8) ml/kg/min. Intermittent exercise (INT) = 11 women: Age = 49 (8); BMI = 32.2 (5.1); VO_2_ max = 22.9 (4.1) ml/kg/min	18 mos	Supervised exercise. CON = 30 min/day, 3 days/wk., at 60–70% max VO_2_. INT = Brisk walk at approximately 50–60% HRR, 2 times/day, 15 min/session, 5 days/wk. at their home or worksite.	EI and macronutrient intake measured with 3-day food records (2 wk days, 1 wk end day) at baseline, 9, and 18 mos. 24-hr recalls were collected at 3, 6, 12, and 15 mos.	Exercise adherence: CON = 90.1 (2.3)%; INT = 90.3 (9.1)%; Supervision: CON = 100%; INT = 17.9 (4.8)%. Weekly EEEx (kJ): CON = 2235 (614); INT = 3235 (872)	Total EI did not change significantly during the duration of the study. Macronutrient composition was unchanged with the exception of fat intake for INT which significantly lower at 9 and 18 mos compared to baseline. Results for the 24-hr recalls were similar to the 3-day food records.
Foster-Schubert *et al.* (2012) [110] Exercise vs. Control Arms	Control = 87 women: Age = 57.4 (4.4); BMI = 30.7 (3.9); Non-Hispanic white = 85.1%; VO_2_ max = 23.1 (4.1) ml/kg/min. Exercise = 117 women: Age = 58.1 (6.0); BMI = 30.7 (3.7); Non-Hispanic white = 83.3%; VO_2_ max = 22.5 (4.1) ml/kg/min. Completion rates: Control = 80/87 (92%); Exercise = 106/117 (91%)	12 mos	Control: no exercise. Exercise: Progressed from 15 min/session, 60–70% max HR, 5 days/wk to a target of 45 min/session, 70–85% max HR, 5 days/wk. at week 7. Supervised at exercise facility 3 days, at home 5 days.	Women's Health Initiative 120-item self-administered. FFQ administered at baseline and 12 mos.	Exercise adherence: Achieved on average 80% of the target 225 min/wk aerobic exercise over the 12 mo trial.	EI and fat intake (%EI) decreased in both control and exercise groups, with no significant between group differences.
Grediagan *et al.* (1995) [126]	High Intensity Exercise = 6 women: Age = 30 (5); BMI = 23.8 (2.3); White = 50%; VO_2_ max = 31.5 (3.8) ml/kg/min. Low Intensity Exercise = 6 women: Age = 31 (6); BMI = 26.2 (1.4); White = 0%; VO_2_ max = 31.3 (3.3) ml/kg/min. Completion rate: High = 67%;Low = 67%	12 wks	Supervised treadmill exercise, 4 days/wk at either low (50% VO_2_ max) or high (80% VO_2_ max) intensity. Duration was adjusted to elicit EEEx of 300 kcal/session	4-day food records at baseline, 6 and 12 wks.	Exercise adherence: All subjects completed the 48 prescribed exercise sessions at the prescribed work rate.	No significant changes in EI or macronutrient composition in either group.
Jakicic *et al.* (2011) [122]	Control (n = 89): 92.1% female; 78.6% white; Age = 44.7 (7.9); BMI = 27.1 (1.7). Moderate dose (n = 82): 90.2% female; 78.9% white; Age = 43.5 (8.8); BMI = 27.1 (1.7). High dose (n = 98): 91.8% female; 81.1% white; Age = 45.0 (8.4); BMI = 27.0 (1.6). Completion rate: ITT analysis, Control = 94%; Moderate dose = 93%; High dose = 90%. All subjects sedentary.	18 mos	Control: no PA intervention. Moderate dose PA: Progressed to 150 min/wk., 55–85% age-predicted max HR, structured, non-supervised PA at wk. 12. High dose PA: Progressed from 100 to 300 min/wk.	Block FFQ	Estimated EEEx (kcal/wk.): Control 6 mos = 1529; 12 mos = 1439; 18 mos = 1460. Moderate dose: 6 mos = 1504;12 mos = 1324; 18 mos = 1147. High dose: 6 mos = 2008; 12 mos = 1685; 18 mos = 1527	No significant group-by-time interaction for changes in EI or percent of energy from fat, CHO or PRO.
Kirkwood *et al.* (2007) [111] Exercise vs. Control arms	Control = 18 women: BMI = 32.5 (4.5). Exercise = 19 women: BMI = 31.6 (3.8). Mean age for total sample = 41(NR). Total sample completion rate = 63.3%. Activity/fitness level = NR	12 wks	Control: no exercise. Exercise: Specific advice to achieve 60 min brisk walking/day.	4-day unweighed food record (3 wk and 1 wk end day) at baseline, 6 and 12 wks.	EEEx estimated by accelerometer increased 150 kcal/day and 203 kcal/day over baseline at wks 6 and 12, respectively.	Control: EI decreased at wks. 6 and 12 vs. baseline. No change in macronutrient intake as a % of EI over 12 wks. Exercise: EI significantly lower than baseline at wk 6. % energy from fat increased from baseline at wk 12. No change in % energy from CHO or PRO.
Miyashita *et al.* (2010) [127]	Walking group = 8 men: Age = 49.1 (3.4); BMI = 29.7 (2.0); VO_2_ max = 27.4 (1.7) ml/kg/min. Jogging group = 7 men: Age = 46.7 (3.2); BMI = 29.6 (0.7); VO_2_ max = 30.7 (2.0) ml/kg/min.	12 wks	Supervised brisk walking or jogging – 30–60 min/day, 3 days/wk., outdoors. Walking = 65–70% age predicted max HR. Jogging = 75–80% age predicted max HR.	3 day weighed food record at baseline and 10 wks.	Exercise adherence: Walking = 75(8)%; Jogging = 74 (11)%. Exercise intensity: Walking = 69 (4)% max HR. Jogging = 78 (1)% max HR.	No significant differences for total EI or macronutrient intake between or within groups at baseline or at 10 wks.
Nieman *et al.* (1990) [112]	Control group = 18 sedentary women: Age = 32.8 (1.4); BMI = 27.8 (0.9). Exercise group = 18 sedentary women: Age = 36 (1.6); BMI = 28.3 (0.7).	15 wks	Brisk walking, 45 min/session, 5 days/wk at 62% VO_2_ max.	7-day food records at baseline, 6 and 15 wks.	90% of sessions were supervised. Intensity = 62 (2)% VO_2_ max.	EI decreased significantly over 15 wks in the exercise group and showed a non- significant increase in the control group. The between group differences were non-significant. No significant group by time interactions for CHO, fat or PRO intake as a % of EI.
Nordby *et al.* (2012) [113] Exercise vs. control groups	Control = 15 men: Age = 31 (2); BMI = 28.0 (0.4); VO_2_ max = 35.7 (1.7) ml/kg/min. Exercise = 17 men: Age = 28 (1); BMI = 28.3 (0.3); VO_2_ max = 38.2 (1.7) ml/kg/min. Completion rate = Control = 75%; Exercise = 85%.	12 wks	Control: no exercise. Exercise: Supervised endurance training, 3–4 sessions/wk., moderate intensity ∼65% HRR and 3–4 sessions/wk. of continuous exercise with intermittent high intensity training intervals at ∼85% HRR, until the 600 kcal were utilized.	Weighed 3-day food records at baseline, 2, 6 and 12 wks.	EEEx = 576.3 (10.9) Kcal/day. Exercise duration = 51.4 (4.3) min/day	No significant between group differences for change in EI, or % of energy from fat, CHO or PRO.
Prichard *et al*. (1997) [114] Control vs. Exercise Arms	Control = 19 men: Age = 42.3 (4.5); BMI = 28.6 (2.8). Exercise = 21 men: Age = 44.9 (6.5); BMI = 29.2 (2.8). Completion rate = 62% of those that started the intervention. Activity/fitness level = NR.	12 mos	Control: no exercise. Exercise: Self-selected to be completed in leisure time; minimum 3–30 min sessions/wk at a recommended intensity of 65–75% of max HR.	3-day food records at 6 and 12 mos. and monthly 24-hr recalls.	Exercise modes: Of the 21 exercisers, 11 walked, 4 jogged (2 alternated jogging with swimming) 3 attended a gym (45 min of aerobic, 15 min RT and anaerobic exercise), and 3 rode an exercise bike. Exercise participation: 3 and 7 sessions/wk.	No significant changes in EI or % energy from fat in either the control or exercise groups.
Reseland *et al.* (2001) [117] Control vs. exercise arms	Control = 37 men. Exercise = 48 men. Mean age (complete sample) = 44.9 (2.5). Mean BMI (complete sample) = 28.6 (3.4). No data or data from which to calculate completion rate are presented. Activity/fitness level = NR	12 mos	Control: no exercise. Exercise: Supervised, 3 day/wk., 60 min/session at 60–80% of peak HR. Sessions were 60% aerobics, 25% circuit training and 15% jogging/fast walking.	FFQ completed at baseline and 12 mos.	NR	Fat intake as a % of EI was significantly lower at 12 mos. vs. baseline in the exercise group; intakes of other macronutrient was unchanged in both exercise and control groups over the 1-year intervention period.
Ready *et al.* (1995) [115]	Control = 10 women: BMI = 32.1 (4.1); VO_2_ max = 24.2 (2.8) ml/kg/min. Walk group = 15 women: BMI = 29.4 (6.1); VO_2_ max = 28.5 (4.9) ml/kg/min. Average age of total sample = 60.9 (4.6). Completion rate: Control = 63%, Walk = 67%.	6 mos	Control: no exercise. Walk: 60 min/day, 5 days/wk at 60% HRR.	3-day food record at baseline and 6 mos.	Estimated EEEx = 6367 (1890) kJ/wk. Duration = 54.3 (7.7) min/session. Intensity = 54% HRR.	No significant change in EI or diet composition.
Ready *et al.* (1996) [116]	Control = 20 women: BMI = 26.6 (3.8); VO_2_ max = 21.8 (2.8) ml/kg/min. 3-day group = 19 women: BMI = 26.8 (4.3); VO_2_ max = 23.2 (4.3) ml/kg/min. 5 day group = 17 women: BMI = 26.2 (2.5); VO_2_ max = 22.3 (4.3) ml/kg/min. Mean age complete sample = 61.3 (5.8). Completion rate = 71% overall. Control group = 80%; 3-day group = 70%; 5-day group = 63%	24 wks	Control: no exercise. Exercise: Walk (outdoor track), 60 min/session at 60% VO_2_ peak either 3 or 5 days/wk. Successful completion required 150 min/wk. and 240 min/wk. for the 3 and 5 day/wk. groups, respectively.	3-day food records (2 wk days and 1 wk end day).	Exercise frequency: 3 day = 2.9 (0.1) days/wk. 5 day = 4.9 (0.4) days/wk. Exercise duration: 3 day = 171(8) min/wk. 5 day = 279 (20) min/wk. Estimated EEEx: 3 day = 4380 kJ/wk. 5 day = 7150 kJ/wk. Supervision: 3 day = 1.1 (0.4) sessions/wk. 5 day = 0.9 (0.3) days/wk.	No significant change in EI, or % energy from fat, CHO or PRO in any group over the 24 wk trial.
Rosenkilde *et al.* (2012) [121]	Control = 17 men: Age = 31 (6); BMI = 28.0 (2.3); VO_2_ max = 35.9 (4.8) ml/kg/min. Moderate dose exercise (MOD) = 18 men: Age = 30 (7); BMI = 28.6 (1.8); VO_2_ max = 34.6 (4.1) ml/kg/min. High dose exercise (HIGH) = 18 men: Age = 28 (5); BMI = 27.6 (1.4); VO_2_ max = 36.2 (5.3) ml/kg/min. Completion rates: Control = 17/18 = 94%; MOD = 18/21 = 86%; HIGH = 18/22 = 82%	13 wks	Control: no exercise. MOD dose: running/cycling 300 kcal/day. Intense sessions (>70% VO_2_ max) 3 days/wk. HIGH dose: running/cycling 600 kcal/day. Intense sessions (>70% VO_2_ max) 3 days/wk.	3-day weighed food records at baseline and wk. 11.Diets for ad libitum consumption were presented over 8 days at baseline and wk 13. Diets were provided in a randomized double-blinded order and consisted of 4 consecutive days of either high-CHO or low-CHO diet, respectively.	Exercise adherence: MOD = 99%, HIGH = 96%. Training EEEx: MOD = 335 (8) kcal/day. HIGH = 653 (10) kcal/day. Exercise duration: MOD = 29.9 (8.2) min/session. HIGH = 55.2(6.6) min/session.	No difference in EI in response to the intervention between or within groups. The intervention did not change the relative contribution of dietary macronutrients between or within the groups. No changes between or within groups were observed when ad libitum EI was expressed as total EI or relative to body weight during a high-CHO or low-CHO diets,
Shaw *et al.* (2010) [118]	Control = 12 sedentary men: Age = 25.0 (2.4); BMI = 24.7 (2.7). Endurance training (ET) = 12 sedentary men: Age = 25 (5.6); BMI = 23.9 (2.3). Concurrent endurance+Resistance training (CT) = 13 sedentary men: Age = 26 (3.1); BMI = 26.8 (5.0)	16 wks	Control: no exercise. ET: Rowing, stepping, cycling and walking on treadmill for 45 min at 60% age predicted max HR, 3 days/wk. CT: 22 min of endurance training as described for the ET group+3 sets/15 reps at 60% 1-RM for 8 exercises. All exercise was supervised.	7-day food records at baseline and 16 wks.	Exercise adherence or EEEx were NR	No association between EI and treatment group. However there was a significant decrease in total EI and in absolute intake (g/day) of CHO, fat and PRO in the CT group.
Van Etten *et al.* (1997) [119]	Control = 8 sedentary men: Age = 35 (6); BMI = 23.6 (NR). Exercise = 18 sedentary men: Age = 33 (6); BMI = 23.8 (NR)	18 wks	Control: no exercise. Exercise: Supervised resistance training, 2 non-consecutive days/wk., 3 sets, 15 reps, 10 exercises.	3-day food record (including 1 wk end day) at baseline 8, and 18 wks.	Exercise compliance = 95 (7)%	EI and macronutrient intake did not differ between baseline, 8 and 18 wks in either control or exercise groups.
Washburn *et al.* (2012) [120]	Control = 13 sedentary men; Age = 20.8 (3.5); BMI = 27.7 (2.5).10 sedentary women: Age = 21.6 (2.5); BMI = 26.4 (3.3). Exercise = 17 sedentary men: Age = 21.1 (2.6); BMI = 27.6 (2.3); 15 sedentary women: Age = 19.1 (1.3); BMI = 27.3 (3.5)	6 mos	Control: no exercise. Exercise: Supervised resistance training, 3 days/wk, 1 set, 9 exercises, 3–6 repetition max.	Monthly–randomly selected period (2 wk days, 1 wk end day) using 24-hr recalls.	Exercise adherence = 96% of scheduled training sessions.	No significant between group differences at baseline or during the intervention for EI or macronutrient intake as a % of EI in the total sample or for men or women.

*Note*, Values are means (standard deviation) unless otherwise noted. Abbreviations: CHO = carbohydrate; PA = physical activity; EEEx = exercise energy expenditure; EI = energy intake; FFQ = food frequency questionnaire; HRR = Heart Rate Reserve; NR = not reported; PRO = protein; REI = relative energy intake; kcal = kilocalories; KJ = kilojoules; MET = Metabolic equivalent of task; hrs = hours; min = minutes; wk = week.

### Randomized Trials: Study Characteristics

#### Sample size/completion rate

The median (range) sample size across the 24 randomized trials was 43.5 (12–411). The median (range) proportion of randomized participants who completed the intervention and provided data for energy intake for the 23 trials that provided data on this parameter was 74% (21–100%).

#### Trial length

The median (range) length of the 24 randomized trials was 21 (12–72) wks.

#### Exercise mode

Eight of 24 randomized trials involved laboratory based aerobic exercise where participants used a variety of modalities including cycle ergometers, rowers, recumbent cycles, steppers and treadmills [16], [110], [113], [114], [117], [118], [123], 8 evaluated indoor/outdoor walk/jog [107], [111], [112], [115], [116], [122], [124], [127], 5 trials employed primarily laboratory based treadmill walking/jogging [108], [109], [125], [126], [128], 4 trials involved resistance training only [108], [119], [120], [123], 2 trials used a combination of resistance and aerobic training [118], [123] and one trial each involved swimming [124] and laboratory cycle ergometer exercise [129].

#### Exercise supervision

All exercise sessions were supervised in 15/24 (63%) of randomized trials [16], [107]–[109], [112], [113], [117]–[120], [123], [125]–[127], [129], partially supervised in 6 trials (25%) [110], [115], [116], [121], [124], [128] and unsupervised in 3 trials (∼13%) [111], [114], [122].

#### Exercise prescription (frequency)

The median (range) exercise frequency was 4 (2–7) days/wk in trials/groups randomized to aerobic exercise and 3 (2–4) days/wk for participants randomized to resistance training.

#### Exercise prescription (intensity)

Seven randomized trials prescribed intensity as a percentage of maximal VO_2_ [16], [108], [112], [116], [121], [123], [128], 8 trials used a percentage of HRMax [110], [114], [117], [118], [122], [125]–[127], 6 used a percentage of heart-rate-reserve [109], [113], [115], [124], [128], [129] and in 2 trials intensity was “self-paced” [107], [111]. The median (range) of intensity prescriptions were: 65% (50–85%) maximal VO_2_; 70% (50–90%) HRMax, and 65% (50–75%) heart-rate-reserve.

#### Exercise prescription (duration)

Sixteen randomized trials prescribed exercise duration by time [108]–[112], [114]–[118], [122], [124], [125], [127]–[129], 3 by level of exercise energy expenditure [113], [121], [126], and one each by energy expenditure/kg body weight [16], caloric equivalent of walking 12 miles/wk [123] and walking distance [107]. The median (range) duration for the 16 trials prescribing exercise by time was 42 (30–60) min/day. Exercise prescriptions by level of energy expenditure were 300 kcal/day [126], 600 kcal/day [113] and both 300 and 600 kcal/day [121]. Church *et al.* [16] randomized participants to energy expenditure groups of 4, 8 and 12 kcal/kg/wk, while Bales *et al.* [123] assigned participants to a combination of aerobic exercise modes (treadmill, elliptical, cycle ergometer) at a caloric equivalent of 12 miles/wk and Brandon *et al.* [107] prescribed walking 3 miles/session.

#### Compliance with the exercise protocol

Eighteen studies presented data relative to participant compliance with the exercise training protocol [16], [107]–[110], [112], [115], [116], [119]–[121], [123]–[128]. Fifteen trials reported the percentage of exercise sessions attended [(median(range) 95% (74–100%)] [16], [107]–[110], [112], [115], [116], [119]–[121], [123], [126]–[128], while 3 trials compared the prescribed with actual minutes of actual exercise completed. Bryner *et al.* [125] prescribed 40–45 min/day, 4 days/wk and observed 45 min/day, 4.1 days/wk. Cox *et al.* [124] prescribed 90 and achieved 124 min/wk while Nordby *et al.* [113] prescribed 600 kcal/session and achieved 576 kcal/session.

#### Energy and macronutrient assessment

Twelve trials used non-weighed food records over 2 to 7 days [107], [108], [111], [112], [115], [116], [118], [119], [121], [125], [126], [129], 2 used weighed 3-day food records [113], [127], 5 used food frequency questionnaires [16], [110], [117], [122], [124], 3 used a combination of food records and 24 hour recalls [114], [123], [128], and one study each employed repeated 24 hour recalls [120], one study used weigh and measure ad libitum eating over 2 weeks [109], and one study used test meals offered over 8 days [121]. Nine studies assessed energy intake only at baseline and end [16], [110], [115]–[118], [121], [123], [127], 7 studies completed energy intake assessments at 3 time points [111]–[114], [119], [124], [126], and 4 studies at 4 time points [107], [108], [122], [125] and 4 at more than 4 time points [109], [120], [128], [129].

### Randomized Trials: Participant Characteristics

#### Age

The median (range) for age across all randomized trials 43 (25–61) years.

#### Gender

Eleven trials (∼46%) included only women [16], [107], [110]–[112], [115], [116], [124]–[126], [128], 9 men only [108], [113], [114], [117]–[119], [121], [127], [129] and four studies (∼17%) included both men and women [109], [120], [122], [123]; however, only 2 of these studies provided separate result data by gender [109], [120].

#### BMI

The median (range) BMI for participants over the 24 randomized trials was 28.3 (25.0–32.4) kg/m^2^. Thirteen of the 24 randomized trials (∼54%) evaluated overweight participants (i.e. BMI>25 to ≤30 kg/m^2^) [108], [109], [112]–[114], [116], [117], [120]–[122], [124], [126], [127], while the mean sample BMI was classified as obese (i.e. ≥30 kg/m^2^) in 8 trials [16], [107], [110], [111], [115], [123], [128], [129] and normal weight (i.e. BMI≤25 kg/m^2^) in 3 trials [118], [119], [125].

#### Minority status

The median (range) percentage of non-white participants was 19% (0–60%) for the 9 randomized that provided information relative to the racial or ethnic composition of the study sample [16], [107]–[110], [122], [123], [126], [129].

#### Participant activity level

With the exception of the trial by Broeder *et al.* [108], who studied active but untrained participants; all randomized trials were conducted in participants who were sedentary or minimally active at baseline.

### Randomized Trials: Results

#### Energy intake

Eighteen of 24 randomized trials (75%) found no significant change in energy intake in response to exercise training [108]–[110], [113]–[117], [119]–[122], [124]–[129]. Five randomized trials reported significant decreases (∼200–500 kcal/day) in energy intake in response to exercise training [16], [111], [112], [118], [123]. One randomized trial reported a significant increase in energy intake (Brandon 06). However, energy intake increased only in African American, but not white women, where energy intake decreased [107].

#### Macronutrient intake

Eighteen of the 23 randomized trials (∼78%) that provided data on macronutrient intake found no change as a result of exercise training [16], [108], [109], [112]–[120], [122], [124]–[127], [129]. Results from the 5 randomized trials that reported significant changes in macronutrient intake as a result of exercise training were mixed [107], [110], [111], [123], [128]. For example, Brandon *et al.* [107] reported a significant increase in absolute carbohydrate intake in white, but not African American women, while Kirkwood *et al.* [111] reported a significant increase in the intake of fat as a percentage of total energy intake, with no change in the percentage of energy intake from carbohydrate or protein. Studies reported significant decreases in the absolute intake of carbohydrate [107], [118], [123], fat [118], [123], [128] and protein [118], [123] as well as decreases in the intake of fat as a percentage of total energy intake [117].

### Effect of Study Parameters on Energy Intake

#### Exercise mode

Two randomized trials compared change in energy intake in response to aerobic and resistance training. Broeder *et al.* [108] reported no change in energy intake in either the aerobic or resistance training groups while Bales *et al.* [123] reported a significant decrease in energy intake induced by aerobic, but not resistance training. A combination of aerobic plus resistance training was compared with aerobic training alone in 2 trials. Bales *et al.* [123] reported no between group differences for the change in energy intake with significant decreases in energy intake in both groups. Shaw *et al.* [118] reported significant decreases in energy intake in the aerobic plus resistance training group but not the aerobic training group. No significant changes in energy intake were reported in the 2 trials that evaluated energy intake in response to resistance training compared with non-exercise controls [119], [120] or between participants who completed swim vs. walking training programs [124].

#### Level of exercise energy expenditure/duration

No between group differences for change in energy intake in response to aerobic exercise training at difference levels of exercise energy expenditure were reported in the 4 trials that evaluated this parameter [16], [116], [121], [122].

#### Exercise intensity

Four randomized trials compared changes in energy intake in response to aerobic exercise training at low or high intensity [125]–[127], [129]. No significant between group differences for change in energy intake were reported in any of the 4 trials.

#### Intermittent vs. continuous exercise

The one randomized trial that compared changes in energy intake in response to continuous (one-30 minute sessions/day) vs. intermittent exercise (2–15 minute sessions/day) reported no between or within group differences [128].

#### Composition of test meals

Rosenkilde *et al.* [121] found no differences in energy intake with exercise training when low or high carbohydrate test meals were offered.

### Effect of Participant Characteristics on Energy Intake

#### Age

No randomized trials evaluated the effect of age on changes in energy intake in response to exercise training.

#### Gender

Two randomized trials provided data on gender differences for changes in energy intake in response to exercise training [109], [120]. Donnelly *et al.* [109] reported no between group (exercise vs. control) for change in energy intake in response to 16 months of supervised exercise in either men or women. Similarly, Washburn *et al.* [120] found no between group differences (exercise vs. control) for change in energy intake in response to 6 months of supervised resistance training in either men or women.

#### Other participant characteristics

No randomized trials were identified that specifically evaluated the effect of weight status, level of physical activity or level of dietary restraint on the energy intake response to exercise training.

#### Risk of bias

The risk of bias for all randomized trials is presented in Table 5. The description of the procedures for random sequence generation were unclear in the majority of trials (16/24 - ∼67%). Six trials adequately described randomization procedures and were considered low risk of bias [109], [110], [113], [121], [123], [124], while 2 trials were considered high risk for randomization bias based on failure to provide any description of the randomization process [119] or randomization based on level of occupational and lifestyle physical activity [127]. With the exception of the trial reported by Cox *et al.* [124], which adequately described procedures for allocation concealment (low bias) all other randomized trials (96%) provided no description of procedures for allocation concealment. Blinding participants and personnel is not feasible in an exercise trial. Blinding of personnel performing outcome assessments is feasible in exercise trials; however, this was described in only 2 trials [110], [121]. Twenty one trials provided no information relative to blinding of outcome assessments, while one trial directly stated that outcome assessments were not blinded [113]. Based on an effectiveness study paradigm the risk of attrition bias is high in the majority of the 24 randomized trials included in this review. Fifty-four percent of trials reported completion rates of less than 80%; however, 22 of 24 of these studies were conducted as efficacy trials where data from participants who were non-adherence to the exercise intervention or outcome assessment protocols were not included in the analysis.

**Table 5:** Study Risk of Bias.

Study	Random Sequence Generation (selection bias)	Allocation concealment (selection bias)	Blinding participants and personnel (performance bias)	Blinding of outcome assessment (detection bias)	Incomplete outcome data (attrition bias)	Selective reporting (reporting bias)	Other bias
Bales *et al.* (2012) [123]	Low risk	NR	High risk	NR	High risk	Low risk	High risk
Brandon & Elliott-Lloyd (2006) [107]	Unclear	NR	High risk	NR	High risk	Low risk	High risk
Broeder *et al.* (1992) [108]	Unclear	NR	High risk	NR	High risk	Low risk	High risk
Bryner *et al.* (1997) [125]	Unclear	NR	High risk	NR	High risk	Low risk	High risk
Church *et al.* (2009) [16]	Unclear	NR	High risk	NR	High risk	Low risk	High risk
Cox *et al.* (2010) [124]	Low risk	NR	High risk	NR	High risk	Low risk	High risk
Cox *et al.*(2003) [129]	Unclear	NR	High risk	NR	Low risk	Low risk	High risk
Donnelly *et al.* (2003) [9]	Low risk	NR	High risk	NR	High risk.	Low risk	High risk
Donnelly *et al.* (2000) [128]	Unclear	NR	High risk	NR	Low risk	Low risk	High risk
Foster-Schubert *et al.* (2012) [110]	Low risk	NR	High risk	Low risk	Low risk	Low risk	High risk
Grediagin *et al.* (1995) [126]	Unclear	NR	High risk	NR	High risk	Low risk	High risk
Jakicic *et a*l. (2011) [122]	Unclear	NR	High risk	NR	Low risk	Low risk	High risk
Kirkwood *et al.* (2007) [111]	Unclear	NR	High risk	NR	High risk	Low risk	High risk
Miyashita *et al.* (2010) [127]	High risk	NR	High risk	NR	Low risk	Low risk	High risk
Nieman *et al.* (1990) [112]	Unclear	NR	High risk	NR	Low risk	Low risk	High risk
Nordby *et al.* (2012) [113]	Low risk	NR	High risk	High risk	Low risk	Low risk	High risk
Pritchard *et al.* (1997) [114]	Unclear	NR	High risk	NR	High risk	Low risk	High risk
Ready *et al.* (1995) [115]	Unclear	NR	High risk	NR	High risk	Low risk	High risk
Ready *et al.* (1996) [116]	Unclear	NR	High risk	NR	High risk	Low risk	High risk
Reseland *et al.* (2001) [117]	Unclear	NR	High risk	NR	NR	Low risk	High risk
Rosenkilde *et al.* (2012) [121]	Low risk	NR	Low risk	Low risk	Low risk	Low risk	High risk
Shaw *et al.* (2010) [118]	Unclear	NR	High risk	NR	Low risk	Low risk	High risk
Van Etten *et al.* (1997) [119]	High risk	NR	High risk	NR	Low risk	Low risk	High risk
Washburn *et al.* (2012) [120]	Unclear	NR	High risk	NR	Low risk	Low risk	High risk

*Note*. NR = Not Reported.

## Discussion

### Summary of Evidence

In this paper we systematically reviewed 99 studies that employed a variety of study designs including cross-sectional, acute/short-term, non-randomized and randomized trials to address the question: Does increased exercise or physical activity alter ad-libitum daily energy intake or macronutrient composition in healthy adults? Our results can be summarized as follows.

#### Energy intake

It is commonly believed that individuals increase energy intake in response to increased physical activity or exercise training. However overall, we found no consistent, compelling evidence that any level of increased physical activity or exercise has any impact on energy intake. Forty-one percent of cross-sectional studies reported higher energy intake among active compared with inactive individuals. However, cross-sectional data precludes determination of cause and effect. Likewise, it is not possible to determine if the higher energy intake observed among inactive individuals meets or exceeds their level of daily energy expenditure or how between group differences in body weight may impact the results of cross-sectional studies. In agreement with the results of the recent meta-analysis by Schubert *et al.* [30] on acute exercise and subsequent energy intake, our results from both acute and short-term trials suggest that any observed increase in post-exercise energy intake only partially compensates for the energy expended during exercise. Thus, in the short-term, exercise results in a negative energy balance. Results from both non-randomized and randomized trials are in agreement with the results from acute and short-term trials. Only 2 of 36 (∼6%) non-randomized and randomized trials, ranging in duration from 3 to 72 weeks, report an increase in absolute energy intake in response to exercise training, thus implying that exercise does not result in a compensatory increase in energy intake. A limited number of studies across all study designs have evaluated the effect of exercise parameters and participant characteristics on energy intake. Our results suggest no effect of either exercise parameters including mode (aerobic, resistance), intensity, duration/energy expenditure or participant characteristics including age, gender, weight or physical activity level on energy intake. These results are in contrast to those of Schubert *et al.* [30] from their meta-analysis on the acute effect of exercise on absolute post-exercise energy intake where they noted individuals with low to moderate levels of physical activity were more likely to reduce energy intake in response to exercise compared to their more active counterparts.

#### Macronutrient intake

Data on macronutrient intake was reported in 67 of the 99 studies (68%) included in this review. Irrespective of study design we found no consistent evidence for an effect of exercise on macronutrient intake. Forty-four of 54 acute/short-term, non-randomized and randomized trials (81%) reported no effect of exercise on macronutrient intake, while results of the 10 trials reporting an association were mixed. Thus, it appears individuals do not spontaneously alter the composition of their diets in response to physical activity or exercise.

### Limitations in the Available Literature

There are several important limitations in the literature available for this systemic review. The most critical limitation in the available literature is the lack of studies that have been specifically designed and adequately powered to detect significant between or within group differences in energy intake in response to exercise training. With the exception of 2 acute studies [56], [61] no studies included in this review were statistically powered to detect between or within group differences in energy or macronutrient intake. Only 5 of 12 non-randomized trials (∼42%) [97], [100], [102]–[104] and 3 of 24 randomized trials (∼13%) were conducted specifically to evaluate the effect of exercise training on energy and macronutrient intake [112], [121], [125]. The sample size in each of these 3 trials was <20 participants/group. Inadequate statistical power may explain the disconnect between the results of our review, which found no effect of exercise training on energy intake and other trials that have evaluated the effect of exercise on body weight. For example, studies have reported high individual variability in weight loss in response to the same level of exercise energy expenditure [18] and no significant increase in weight loss in response to increased level of exercise energy expenditure [11], [121]. Both of these observations suggest compensatory increases in energy intake in response to exercise training; however, changes in resting metabolic rate and non-exercise physical activity may also play a role.

Because most exercise training studies did not prescribe exercise by level of energy expenditure, assess exercise energy expenditure, or employ adequate methods for the assessment of energy intake, it is not possible to determine the level of exercise induced energy imbalance actually achieved. Only 7 of the 36 non-randomized and randomized trials included in this review (∼19%) prescribed exercise by level of energy expenditure [18], [98], [103], [104], [113], [121], [126] while only 3 of those trials included assessments of the actual level of exercise energy expenditure by indirect calorimetry [18], [104], [126]. Furthermore, the heterogeneity of exercise/physical activity prescriptions prohibits the identification of a specific level that may elicit compensatory changes in energy intake. Only 5 of 36 non-randomized and randomized trials employed more precise estimates of dietary intake such as weigh and measure test meals [18], [103], [104], [121] or observed weigh and measure ad-libitum eating [109]. Two of the 3 trials that measured exercise energy expenditure used only 1 day assessments of energy intake using test meals at baseline and end study [18], [104], while one trial employed 4 day food records at baseline, mid and end study [126]. Thus, reported estimates of energy balance should be cautiously interpreted. The available literature is also limited by a preponderance of data from acute/short-term studies as compared to non-randomized and randomized longitudinal trials. For example, approximately 50% of studies reviewed evaluated the acute or short-term (2–14 days) effect of exercise on energy and macronutrient intake. While acute/short-term studies have employed precise methods for the assessment of energy intake (weigh and measure test meals), the short time frame over which energy intake was assessed may be insufficient to demonstrate significant adaptations in energy intake that may be induced by changes in parameters such as aerobic fitness, body weight or hormonal status etc. associated with longer term exercise training. For example, in overweight men and women Kirk *et al.* [130] have shown that changes in aerobic fitness and weight takes 4 months and does not level off until 9 months in an exercise program with a typical progressive exercise protocol

Approximately 60% of acute studies reviewed recruited participants who were relatively young (median age = 23 years), normal weight, and physically active or aerobically fit. Thus, results from acute trials do not generalize to the older, sedentary overweight population in need of weight management. The available literature is also limited by an insufficient number of studies that have evaluated the impact of exercise parameters (e.g. mode, frequency, intensity, duration, time of day, time course) or participant characteristics (e.g. age, gender, ethnicity, weight, activity level) on energy and/or macronutrient intake.

### Limitations of this Review

Our conclusions should be cautiously interpreted as they are based on both data from sub-optimal study designs (e.g. cross-sectional, acute/short-term, non-randomized trials) and from randomized trials with a high risk of one or more forms of bias. In addition, we did not contact authors to obtain missing data or for clarification of any information presented in the published reports; therefore missing information may reflect reporting bias as opposed to any limitations in the conduct of the study.

## Conclusions

The present systematic review found limited evidence to suggest that acute exercise or exercise training has a significant effect on energy or macronutrient intake. However, as previously discussed the available literature on this topic suffers numerous methodological shortcomings. Therefore, we recommend additional randomized trials to specifically evaluate the impact of exercise training on energy and macronutrient intake that: 1) are powered specifically to detect clinically significant differences in energy and/or macronutrient intake; 2) utilize state-of-the-art techniques for the assessment of energy intake such as direct observation weigh and measure or picture-plate-waste and provide multiple measures across the duration of the study; 3) include assessments of exercise energy expenditure across the duration of the study; 4) evaluate and compare levels of exercise for weight management currently recommended by governmental agencies or professional organizations such as the International Association for the Study of Obesity, the Institute of Medicine, and the American College of Sports Medicine (i.e. 60–90 min/day, moderate intensity) to determine differential effects on energy and macronutrient intake; 5) include overweight and obese, sedentary middle-age or older adults; and 6) evaluate both the effect of exercise parameters (e.g. mode, frequency, intensity duration, time course) and participant characteristics (e.g. age, gender, body weight, activity level, ethnicity) on the association between exercise and energy and macronutrient intake.

## Supporting Information

Checklist S1 **PubMed complete search strategy.**
(DOC)
